# Stable isotopes and predation marks shed new light on ammonoid habitat depth preferences

**DOI:** 10.1038/s41598-021-02236-9

**Published:** 2021-11-23

**Authors:** Marcin Machalski, Krzysztof Owocki, Zofia Dubicka, Oksana Malchyk, Weronika Wierny

**Affiliations:** 1 Institute of Paleobiology, Polish Academy of Sciences, Twarda 51/55, 00-818 Warsaw, Poland; 2 Faculty of Geology, University of Warsaw, Al. Żwirki i Wigury 93, 02-089 Warsaw, Poland; 3 Polish Geological Institute–National Research Institute, Rakowiecka 4, 00-975 Warsaw, Poland

**Keywords:** Evolution, Ecology

## Abstract

Ammonoids are extinct cephalopods with external shells which predominated in many late Paleozoic and Mesozoic marine ecosystems. Stable isotope data from ammonoid shells constitute primary tools for understanding their palaeohabitats. However, in most sedimentary successions globally the aragonitic shells of ammonoids are dissolved during fossilisation process and therefore not available for geochemical studies. We overcome this taphonomic bias by analysing the better preservable calcitic elements of the ammonoid jaws (aptychi). We study moulds and aptychi of two successive members, temporal subspecies in our interpretation, of a scaphitid evolutionary lineage from a Late Cretaceous chalk succession in Poland. In order to reconstruct their habitat depth preferences, we apply the powerful combination of stable isotope data from aptychi and co-occurring benthic and planktic foraminifera with an analysis of predation marks preserved on scaphitid specimens. On this basis we conclude that the populations of the older subspecies led a nektic, and those of the younger subspecies, a nektobenthic lifestyle. The shift in habitat depth preferences took place probably as a response of local populations to the shallowing of the sea. Previous studies largely assumed stable depth preferences for ammonoid species, genera and even higher clades. Our study casts doubts over such generalizations by pointing out that ammonoids could have been more flexible in their depth-related behaviour than anticipated.

## Introduction

Ammonoids rank amongst the most diverse, abundant and best studied clades in the history of life^1–3^. These extinct cephalopods with external chambered shells predominated in many late Paleozoic and Mesozoic marine ecosystems. They followed a pelagic mode of life^4^, inhabiting the water column either close to the sea floor as nektobenthos, or higher up as nekton or plankton. Ammonoid depth preferences may be reconstructed based on several methods, including comparative morphology of conchs, mechanical properties of shell material, and facies inferences^4–8^. The use of stable isotope thermometry of aragonitic ammonoid shells has yielded particularly promising results for depth inferences^9–12^. However, this method excludes from study those specimens that originate from carbonates in which the aragonitic shells were dissolved during fossilisation proces, leaving only natural moulds (steinkerns). This is just the case in the white chalk facies, which predominated in the Boreal Chalk Sea of Europe during the Late Cretaceous^13–15^.

Absence of original shells does not mean, however, that the European chalk ammonoid faunas^16,17^ are completely out of reach where isotope palaeothermometry is concerned. The chalk locally yields aptychi, that is paired calcitic coverings of the lower ammonoid jaws^18,19^. According to Kruta et al.^20^, aptychi were secreted in equilibrium with ambient sea water and are thus potential archives of palaeobiologically meaningful isotope data.

**Figure 1:**
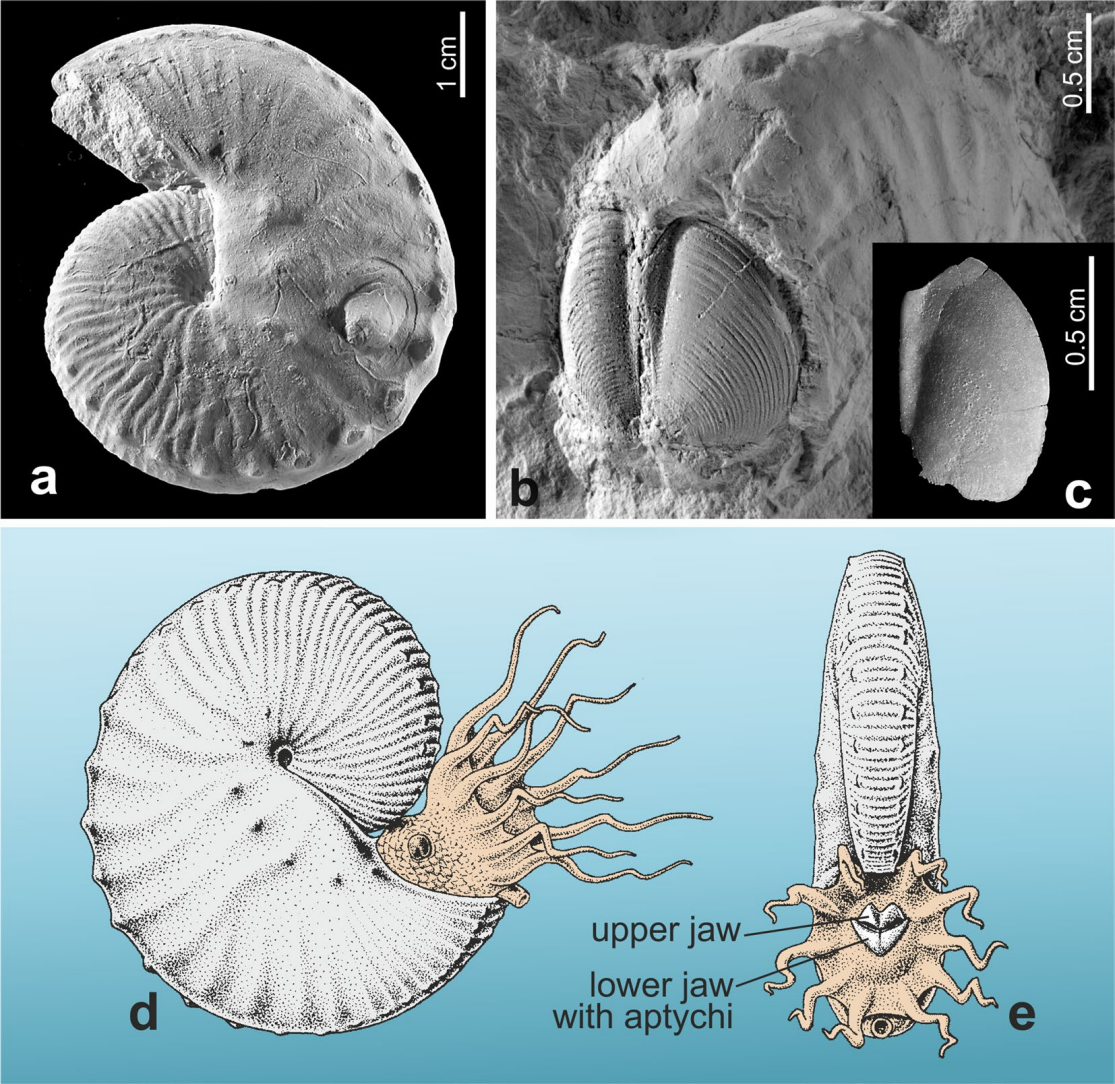
The scaphitid ammonoid studied. (**a**) Mould of *Hoploscaphites constrictus lvivensis* in lateral view (holotype of the subspecies, ZPAL Am. 12/1051). (**b**) A pair of aptychi in internal mould preservation (with discernible growth increments present on the dorsal surfaces of the original aptychi) inside a mould of *H. c. lvivensis*, ZPAL Am. 12/796 (see Machalski, 2021^22^ for interpretation of this specimen and for terminology of aptychi). (**c**) Single aptychus, ZPAL Am. 24/104, in ventral view. (**d**, **e**). Speculative life restoration of the animal in lateral (**d**), and anterior (**e**), views. Specimens (**a**–**c**) from Chełm.

Following the pionieering study by Kruta et al.^20^, we use stable isotope thermometry of aptychi for the reconstruction of habitat depth preferences of a scaphitid, that is member of the ammonoid family Scaphitidae, from the European Boreal Chalk Sea. We target *Hoploscaphites constrictus* (Fig. 1), a species common in the Maastrichtian of Europe^21,22^. More specifically, we study two successive members (which we interpret as two temporal subspecies, or chronosubspecies) of the *Hoploscaphites constrictus* evolutionary lineage^21^, based on specimens and samples from three intervals in the shallowing-upwards epicontinental chalk succession of late Maastrichtian age exposed at Chełm, Poland^23,24^. In order to reconstruct the preferred position of these ammonoids in the water column, we compare temperatures calculated from the oxygen isotope compositions of aptychi with those of co-occurring foraminifera with known bathymetric preferences; we also study carbon isotopes for additional clues^11^.

As an independent test of palaeothermometric inferences, we study predation marks preserved on the scaphitid moulds from Chełm^25^. Quantitative proportions between the various types of such marks in fossil assemblages provide insights into the depth preferences of the ammonoid prey^26,27^.

Habitat depth preferences are an important issue in the discussions on the ammonoid palaeobiology^4,5,8,10,28^. Our results shed a new light on this issue by documenting a shift in depth preferences which took place between closely related members (successive chronosubspecies) of a single lineage of these cephalopods.

## Geological setting

**Figure 2:**
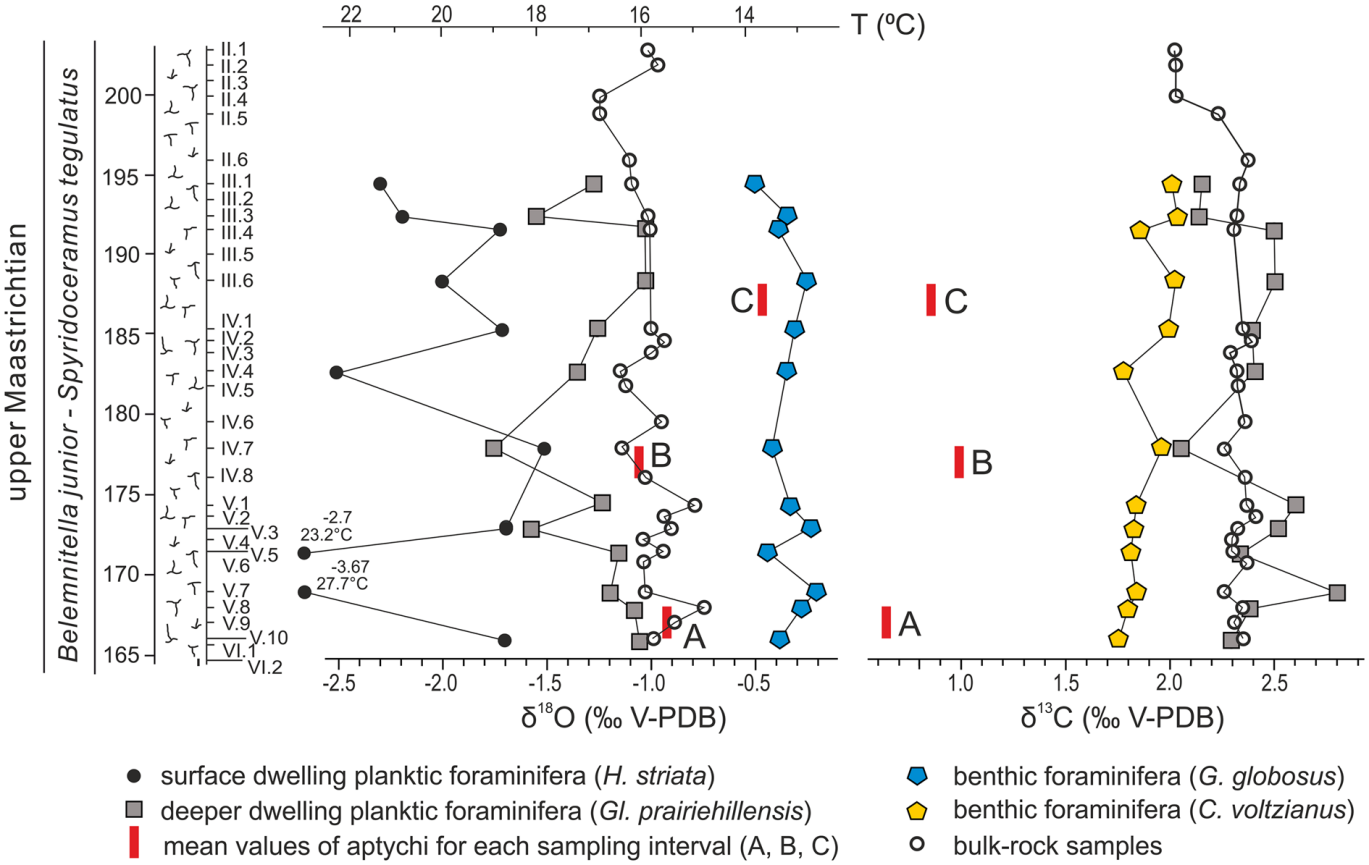
The chalk section at Chełm, with quarry levels VI–II, sampling intervals A–C, and stratigraphic variations in δ^18^O and δ^13^C values of benthic and planktic foraminifera and bulk-rock samples. Red bars are mean δ^13^C and δ^18^O values of aptychi of *Hoploscaphites constrictus* from intervals A–C. *H.*, *Heterohelix*; *Gl.*, *Globigerinelloides*; *G.*, *Gyroidinoides*; *C.*, *Cibicidoides*.

Detailed data on geological setting are provided in the Supplementary Information (SI). Briefly, our specimens and samples come from the 40-m-thick chalk succession exposed at the cement-plant quarry of Chełm, Poland^23^ (Fig. 2; SI-Figs. 1, 2). Due to peculiarities of chalk excavation in the mine (see SI), our sampling of macrofossils, including scaphitid moulds and aptychi, was restricted to three chalk intervals, A–C, each 2 m thick (Fig. 2, SI-Fig. 1).

During the Late Cretaceous, the present-day site of Chełm was located in the eastern part of the the Boreal Chalk Sea of Europe (Surlyk et al.^13^; Thibault et al.^14^; Wilmsen and Niebuhr^15^). The Chełm chalk was laid down in an epicontinental (epiplatform) setting and was separated from the classical areas of chalk sedimentation in Denmark and northern Germany by an area in which sedimentation of spiculitic limestones, the so-called opokas, prevailed (e.g., Leszczyński^29^; Jurkowska and Świerczewska-Gładysz^30^). The current coordinates of the Chełm locality are 51.1303° N, 23.5303° E. According to GPlates Web Service^31^, the palaeo-position of this site 69 myr ago was 47.77° N, 22.16° E. During the Maastrichtian, this area witnessed a progressive cooling trend with lower and higher temperature fluctuations^14^.

The Chełm chalk is assigned to the lower upper Maastrichtian *Belemnitella junior* and *Belemnitella junior-Spyridoceramus tegulatus* zones of the standard European Boreal subdivisions^21,23,32^. Based on correlation with the Stevns-1 reference core in Denmark (SI-Fig. 2; Surlyk et al.^33^; Thibault et al.^14^), we approximately date intervals A, B, and C as 69.0, 68.7, and 68.3 Ma, respectively. The Chełm chalk matches the characteristics of the ‘benthos-poor chalk’ in the Boreal Chalk Sea facies model^13^. Planktic foraminiferal assemblages point to sea level fall during chalk deposition, from a depth of c. 100 m for interval A, to that of several dozen metres for interval C (see Dubicka and Peryt^23,24^, also SI). The shallowing upwards trend for the Chełm succession is confirmed by REE data (SI). Benthic foraminifera assemblages^23^, and macro- and trace-fossils (SI) testify to oxic bottom conditions and normal water salinity during deposition of the chalk. We also failed to find pyrite framboids in the sediment which would potentially indicate temporal dysoxia during deposition of the chalk (see SI, compare Tagliavento et al.^34^).

## Material assessment

The material studied has been identified and assessed along several lines of evidence in terms of its suitability to the present study (see SI, and SI-Figs. 3–11); a brief summary follows below.

**Figure 3:**
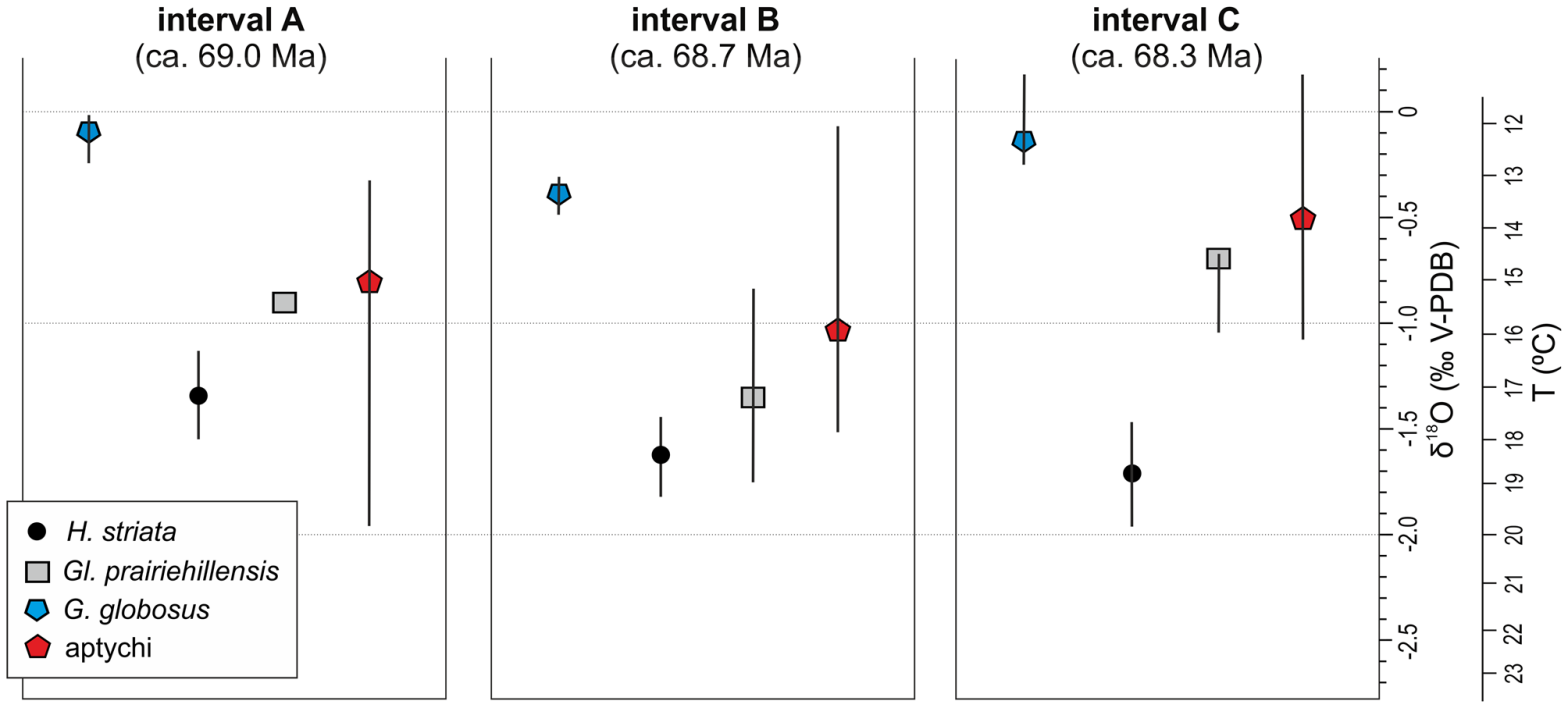
Box and whisker plots of δ^18^O data for targeted foraminifera and aptychi of *Hoploscaphites constrictus* from intervals A–C at Chełm, grouped by taxon. Each box represents the mean value and whiskers are extended between the maximum and minimum δ^18^O values. *H.*, *Heterohelix*; *Gl.*, *Globigerinelloides*; *G.*, *Gyroidinoides*.

**Figure 4:**
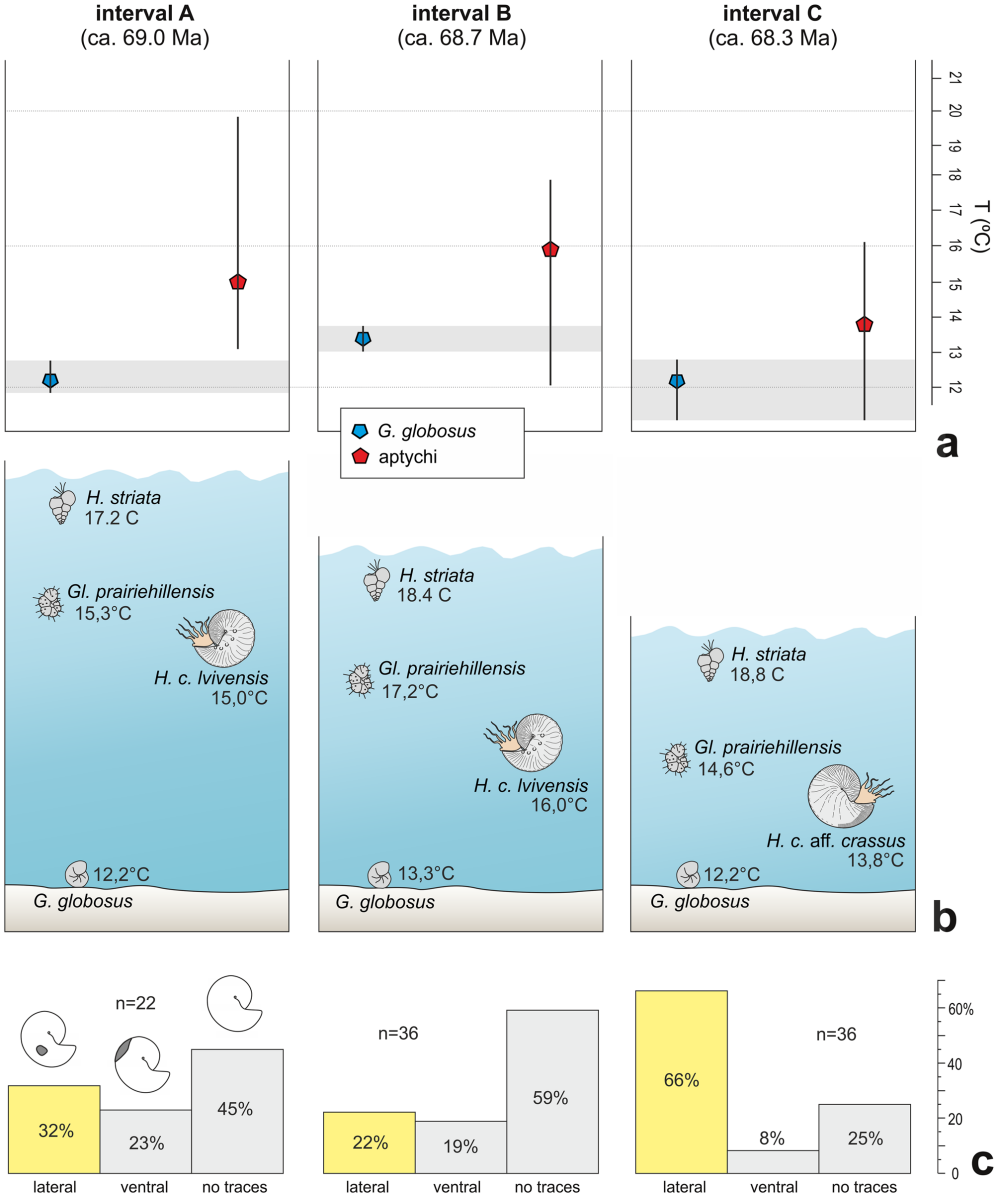
A combination of stable isotope and palaeotemperature data from aptychi of *Hoploscaphites constrictus* and foraminifera with data on the predation marks preserved on moulds of *H. constrictus*, intervals A–C, Chełm section. (**a**) Box and whisker plots of temperature gradient traversed by the scaphitids studied (based on δ^18^O data from aptychi). Each box represents the mean value and whiskers are extended between the maximum and minimum temperature values. Grey shaded areas represent putative bottom waters gradient based on δ^18^O data from the benthic foraminifer *G. globosus*. (**b**) Positions of taxa studied in the water column of the shallowing Maastrichtian sea, based on mean palaeotemperatures from δ^18^O data. (**c**) Incidence of predation traces on *H. constrictus* (see SI-Fig. 8). *H.*, *Heterohelix*; *Gl.*, *Globigerinelloides*; *G.*, *Gyroidinoides*; *C.*, *Cibicidoides*; *H. c*., *Hoploscaphites constrictus*.

**Figure 5:**
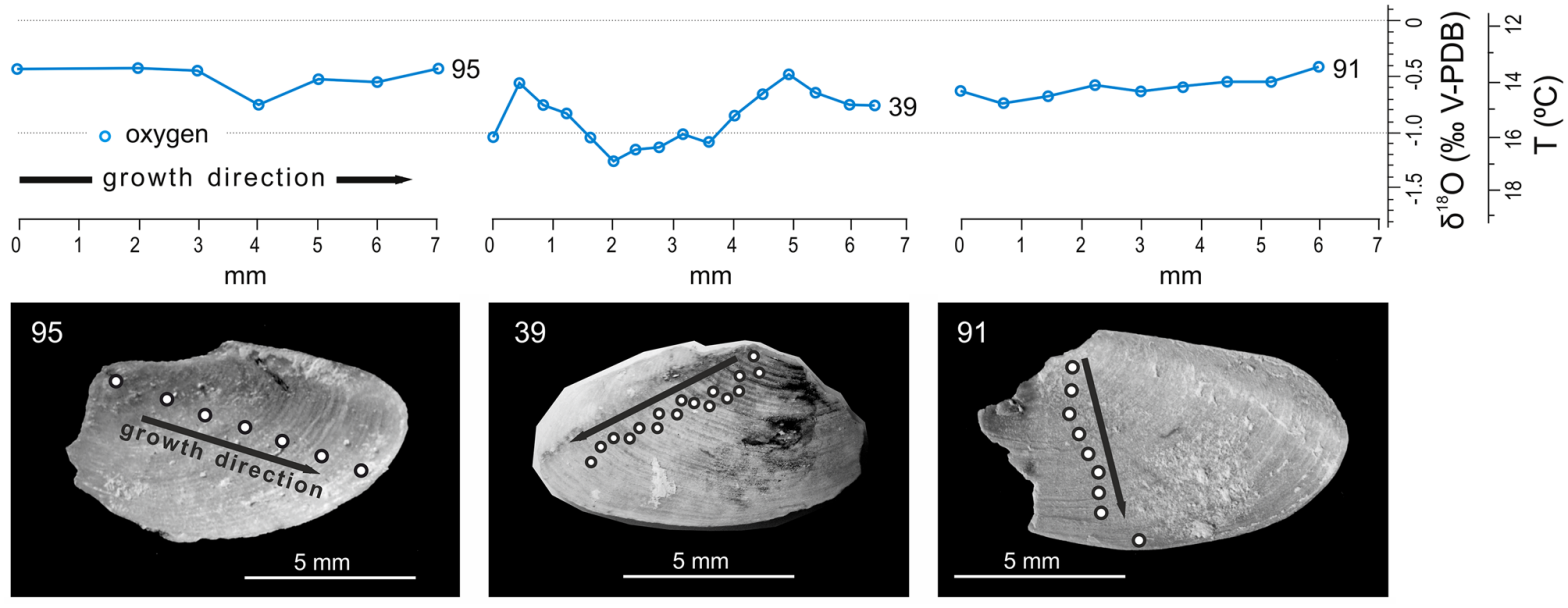
Examples of ontogenetic δ^18^O variation in aptychi of *Hoploscaphites constrictus* (from left to right: ZPAL Am. 24/95 from interval A, 39 from interval B, and 91 from interval C), photographed from their concave, anatomically dorsal, side which reveals growth increments (compare Machalski, 2021^22^); white circles denote sampling points.

**Figure 6:**
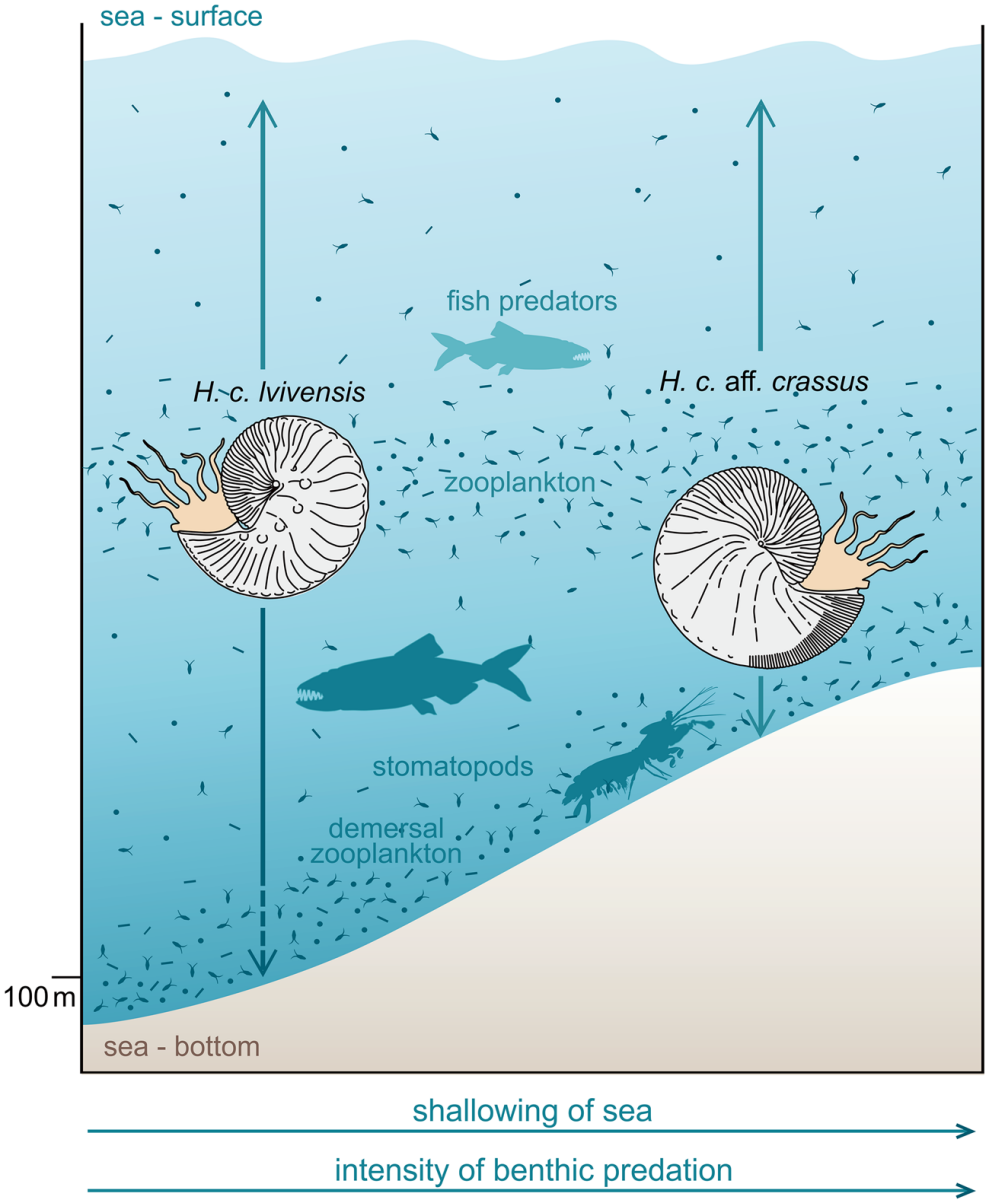
The inferred shift in habitat depth preferences of *Hoploscaphites constrictus* along with shallowing of the sea at the Chełm site*.* See text for further explanations.

The studied scaphitids represent the late Maastrichtian part of the *Hoploscaphites constrictus* evolutionary lineage (SI-Fig. 3). In terms of the subdivison of this lineage into temporal subspecies based on materials from several regions of Europe (SI-Figs. 3–4; Machalski^21^), the scaphitid moulds and co-occurring aptychi (Fig. 1a–c) from intervals A and B at Chełm are assigned to *H. c. lvivensis*, and those from C to *H. c.* aff*. crassus*, intermediate in shell ornament to *H. c. crassus* (SI-Figs. 5–7). The latter subspecies was interpreted by Machalski^21^ as a direct descendant of *H. c. lvivensis*; the presence of the transitional form at Chełm provides an additional argument for this interpretation.

*Post-mortem* transport of the scaphitid remains from distant habitats is ruled out on taphonomic grounds. Specifically, the in situ character of the assemblages studied is indicated by occurrences of aptychi in scaphitid body chambers (SI-Fig. 7), the common presence of double-valved aptychi (SI-Fig. 6), as well as the occurrence of carbonised remnants of the originally chitinous lower jaws still attached to aptychi (SI-Fig. 6).

The pristine preservation documented on microstructural and geochemical grounds for aptychi (SI-Figs. 9–10) and foraminifera^35^ make them suitable for palaeobiologically oriented geochemical analyses. Firstly, the lack of diagenetic alteration of the aptychi is indicated by the presence of well-defined increments and growth lines (SI-Fig. 9) and pristine lamellar microstructures with distinct tablets without any signs of diagenetic secondary phases (SI-Fig. 9). This means a high Preservation Index 5 (excellent preservation) of the aptychi studied^20^. Secondly, most of the aptychi examined by us with Cathodoluminescence (CL) microscopy show no luminescence (SI-Fig. 10), indicating the absence of diagenetic alteration. Thirdly, the results of Electron Microprobe Analyses (EMPA) suggest trace element Mn, Sr, Ba content to be below the detection limit of the microprobe (SI Table 2), which is characteristic for diagenetically unaltered samples. Fourthly, the ontogenetic stable isotope profiles of aptychi from intervals A–C commonly reveal oscillating values of δ^18^O and δ^13^C (SI-Figs. 15–16) which may be interpreted as reflecting original seasonal biological cycles; diagenesis tends to homogenise such signals.

Among foraminifera selected for analyses (SI-Fig. 11), there are two benthic species, *Gyroidinoides globosus* and *Cibicidoides voltzianus*, and two planktic ones, surface-dwelling *Heterohelix striata* and deeper-dwelling *Globigerinelloides prairiehillensis*. Of these, *G. globosus*, *H. striata* and *G. prairiehillensis* precipitated oxygen, and *C. voltzianus* and *G. prairiehillensis* carbon isotopes in near-equilibrium with ambient water^35^. Thus, these foraminifera are expected to yield reliable data for reconstructing the depth-related temperature profile of the water column, against which the scaphitids may be positioned via isotope data from aptychi.

## Results

### Stable isotope analyses

Our isotope analyses are based on bulk rock and foraminiferal samples from the entire section and bulk and serially sampled aptychi from intervals A–C. We used isolated, i.e. found loose in the chalk, aptychi for these analyses. The oxygen (δ^18^O) and carbon (δ^13^C) isotope analyses of the calcite and calculated palaeotemperatures for the entire section are presented in Fig. 2, and for intervals A–C in Figs. 3, 4a,b and 5, and SI-Figs. 12–16 (see SI Tables 3–13 for rough data).

In order to identify significant differences in δ^18^O and δ^13^C isotope values between intervals A–C, we perform the non-parametric Kruskal–Wallis test (for each foraminiferal species separately and the bulk-sampled aptychi). Only δ^18^O isotope signatures for aptychi are significantly different (Kruskal–Wallis test, Hc = 14.73, *p* = 0.0006) with heavier oxygen signatures in specimens from interval C (Mann–Whitney pairwise comparisons). Changes in values of δ^13^C show a negligible variance for aptychi and *C. voltzianus* (Kruskal–Wallis test, Hc = 4.989, *p* = 0.0827; Hc = 6.433, *p* = 0.0404, respectively) with the former having slightly lighter δ^13^C for interval A and the latter having heavier δ^13^C for interval C. We also test the oxygen isotope variation through ontogeny in some aptychi from intervals A–C (Fig. 5; SI-Figs. 15–16) and find no significant differences between these intervals.

### Predation marks

Two types of lethal injuries inflicted by durophagous predators are discernible on the moulds of *Hoploscaphites constrictus* from Chełm (SI-Fig. 8; see also Machalski and Malchyk^25^). The ventral injuries are represented by subcrescentic or V-shaped notches on the ventrolateral sector of the shell, predominantly near the base of the body chamber. The lateral injuries are represented by subcircular to irregular holes on the flanks of the body chamber. The frequency of these two types of predation marks fluctuates between the sampled intervals, with a marked increase in abundance of lateral traces in interval C (Fig. 4c). The ratio of lateral to ventral traces in the sampled intervals equals 1.16 for interval A, 1.39 for B, and rises to 8.25 in C (SI Table 1). There is no clear relationship between types of predation marks and their frequency on ammonoid shells in the sampled intervals (Kruskal–Wallis test, Hc = 1.143, *p* = 0.56).

## Discussion

The occurrence of exclusively stenohaline micro- and macrofauna throughout the Chełm section (see Geological setting section above and SI) allows us to exclude salinity changes as a factor controlling our oxygen stable isotope results; therefore we interpret these results in terms of palaeotemperatures.

The positions of *Heterohelix striata*, *Globigerinelloides prairiehillensis* and *Gyroidinoides globosus* in the water column reconstructed here on the basis of the oxygen-derived temperatures (Fig. 4a,b) are consistent with the inferred habitat depths of these foraminifera^24,35^ The thermal gradient between the surface and bottom of the water column may be estimated at 4–5 °C, based on the temperature differences between the surface *H. striata* and benthic *G. globosus.* In all intervals studied, palaeotemperatures calculated from the aptychi locate *Hoploscaphites constrictus* in a position between the deeper-dwelling planktic *G*. *prairiehillensis* and the benthic *G. globosus*, closely approximating the latter in interval C (Fig. 4a,b). Statistical testing indicates a significant shift for scaphitid aptychi towards heavier oxygen values in C, suggesting a change of habitat into cooler, deeper waters, closer to the bottom than in A and B. No significant changes in thermal gradient between the samples from intervals A–C are recorded. There is a non-significant shift of dissolved inorganic carbon towards heavier δ^13^C values in the aptychi from interval C, which we link to the shallowing of the sea (the δ^13^C_DIC_ values tend to increase upwards the water column and towards the shore^11^).

It is important to note that the isotope results from bulk-sampled aptychi are averages which reflect the net sum of movements of particular individuals throughout the water-column during the sampled periods. Therefore, these results indicate the most commonly adopted (preferred) positions of these animals within the water-column. The lack of significant directional trends in the δ^18^O (and δ^13^C) isotope values among serially sampled aptychi suggests that the habitat depth of their owners did not significantly change since the later juvenile stages (no records of the earlier stages are available due to damage of the apical portions of all analysed specimens, Figs. 1c, and 5 and SI-Figs. 6 and 9).

In summary, the palaeotemperatures gained from aptychi (Figs. 3 and 4a,b) suggest that *H. c. lvivensis* from intervals A and B shared the same ecozone with the deeper-water planktic foraminifer *G*. *prairiehillensis*, while *H. c.* aff. *crassus* from interval C was very close in its habitat to the benthic *G*. *globosus*. On this basis, a predominantly nektic mode of life may be inferred for *H. c. lvivensis* and a nektobenthic (demersal) one for *H. c.* aff. *crassus* (Fig. 6). This habitat shift must have occurred sometime between deposition of intervals B and C, i.e., between 68.7, and 68.3 Ma. These inferences can be verified by data on predation marks. Following Klompmaker et al.^36^, we interpret the ventral damages as inflicted by predatory fish or coleoid cephalopods. These animals could follow either nektic or nektobenthic life, and therefore their traces are equivocal for bathymetry. The lateral marks were interpreted by Machalski and Malchyk^25^ as left by predatory swimming crabs, following Fraaye^37^. Here, we prefer another interpretation—that the lateral injuries result from predatory activity of stomatopods, which smashed ammonoid shells with their raptorial appendages^26^ (see SI for discussion). These crustaceans are exclusively benthic animals^26,38^. Therefore, the major increase in abundance of their traces on *H. c.* aff. *crassus* moulds from interval C (Fig. 4c, SI Table 1) confirms the demersal mode of life inferred for this subspecies from isotope thermometry (Fig. 6).

The depth preferences of *Nautilus*, the only Recent analogue of extinct ectocochleate cephalopods, vary between populations, and are controlled by a variety of factors, including feeding preferences, temperature, and requirements for buoyancy regulation^39^. *Nautilus* is an active scavanger moving several hundred meters up and down along reef slopes, and its maximum depth range (over 700 m) is related to shell strength preventing it from implosion under water pressure^40^. In contrast, scaphitids are thought to have been rather sluggish, mostly demersal swimmers or occassional passive floaters, feeding on zooplankton and confined to shallow-marine milieus by virtue of their thin shells^41,42^. More specifically, Tsujita and Westermann^43^ calculated maximum habitat depths for Campanian and Maastrichtian scaphitids from Canada based on septal strength, suggesting that most of these species could not venture below depths of 100 m. For their *Hoploscaphites* sp. α, which is most closely similar in shape and size to *H. constrictus*, these authors estimated a maximum habitat depth of c. 70 m. By analogy, we posit broadly similar depth restrictions for European scaphitids. According to our estimations, basin depth at Chełm ranged from 100 m for interval A to several dozens of metres for C (see SI for discussion). We therefore propose that during deposition of the chalk intervals A and B (69.0, and 68.7 Ma), the sea floor was located near the maximum habitat depth of *Hoploscaphites constrictus* (Fig. 6). The majority of individuals spent most of their time high in the water column, living within and feeding on zooplankton ‘clouds’^44^. That some individuals occasionally descended to the bottom is evidenced by traces of stomatopod predation on *H. c. lvivensis* from intervals A and B. The situation was different during deposition of the shallowest chalk interval C (68.3 Ma). At this time, *H. constrictus* aff. *crassus* populations followed a demersal life style, conducting massive exploration of the sea floor. They presumably fed here mainly on demersal zooplankton, that is mobile, benthic organisms, which periodically move up into the water column^45^. At the same time, the scaphitids more often fell prey to benthic stomatopods operating on the sea floor (Fig. 6).

The inferred change in lifestyle was associated with the replacement of *Hoploscaphites constrictus lvivensis* by another member of the evolutionary lineage, identified as *H. c*. aff. *crassus*. Therefore, it is tempting to explain this shift in terms of evolutionary process. However, our preferred hypothesis is that it reflects just an opportunistic, reversible response of local populations to new demands of local environment. Based on the Walter’s Law of Facies, we expect that each of our samples A–C had its counterparts in bathymetrically different zones in other parts of the chalk basin. We hypothesise that the scaphitid populations from these zones led different mode of life than their Chełm equivalents. Testing of this hypothesis is, however, not possible with the data at hand. The Chełm section is the only one in Europe which allows for adequate sampling of this segment of the *H. constrictus* evolutionary lineage. Outside the Chełm quarry, *H. c. lvivensis* was identified only in the environs of Lviv, western Ukraine^21^. However, the outcrops that yielded these materials do not exist today, and only inadequately localised mould specimens and a few aptychi are available in the museum collections, which are unsuitable for a study like the present one.

## Conclusion

Stable isotope data from ammonoid shells constitute primary tools for understanding of their palaeohabitats (e.g., Moryia^10^). However, in most sedimentary successions globally the aragonitic shells of ammonoids are dissolved during fossilisation process and therefore not available for geochemical studies. Following Kruta et al.^20^, we overcome this taphonomic bias by analysing the better-preservable calcitic elements of the jaws (aptychi) of a Late Cretaceous scaphitid species, *Hoploscaphites constrictus*, from a white chalk succession deposited in the Boreal Chalk Sea of Europe.

In order to study the depth preferences of *H. constrictus*, we applied the unique and powerful combination of stable isotope data from aptychi and co-occurring foraminifera with well established depth preferences with an analysis of predation marks preserved on scaphitid specimens. On this basis we infer a change in habitat depth preferences between two successive temporal subspecies of the *Hoploscaphites constrictus* lineage. This change took place probably in response to the shallowing of the sea documented for the succession studied (Fig. 6).

Our results have implications for understanding of ammonoid palaeobiology. As pointed out by Moryia^28^, a fundamental knowledge about habitat depth of ammonoids is crucial for understanding of their palaeoecology and mechanisms of evolution and extinction. Many previous studies assumed, either explicitly or implicitly, stable habitat depth preferences for particular ammonoid species, genera and even higher clades (e.g., Westermann^5^ and Moriya^28^), although the latter author mentioned a single case of reversal of depth preferences in one lineage, namely the Perisphinctoidea. As far as the scaphitids are concerned, these cephalopods are generally regarded to have been demersal animals^8,41^. Our study casts doubts over such generalizations, suggesting that ammonoids could have been more flexible in their depth-related behaviour than previously anticipated. Future studies, preferrably based on densely-spaced and stratigraphically well-constrained samples, are encouraged to explore this important issue in more detail.

## Material and methods

### Material studied

In total, 187 moulds of *Hoploscaphites*, 130 aptychi and 34 bulk-rock samples, plus a number of other macrofossils from Chełm have been collected and analysed. The material was collected by sampling of the entire section, with working levels II–VI as reference horizons, and from detailed sampling of three chalk intervals A–C, each 2 m thick (Fig. 2). Due to the way in which the chalk is excavated here, only these intervals made it possible to collect macrofossil samples that were sufficient for the present study. The macrofossil material is housed at the Institute of Paleobiology, Polish Academy of Sciences (PAS), Warsaw (abbreviated ZPAL Am. 12 and 24). Microfossil samples are stored at the Faculty of Geology, University of Warsaw. Ammonoid specimens from Chełm described by Machalski^21^ have also been studied for comparison.

### Preparation of macrofossils

Specimens were prepared, both in the field and laboratory, using standard mechanical methods: hammer, chisel, needles, and a vibrotool (Paleotools, ME-9100).

### Processing of foraminiferal samples

For carbon and oxygen isotope analyses of foraminiferal tests 34 bulk samples of circa 0.2 kg weight were processed. The samples were mechanically disintegrated in tap water, cleaned in an ultrasonic bath and washed through a 125 micron mesh. This procedure was repeated until obtaining foraminiferal tests completely devoid of infill. From each sample residue, adult, large-sized foraminiferal specimens were hand picked to microcentrifugal tubes. Each taxon (selected for specific ecological preferences and very weak vital effect, see chapter Selection of foraminifera in Supplementary Information) was picked separately to obtain foraminiferal monospecific material of a weight in excess of 2 µg.

### Processing of aptychi and bulk-rock samples

For bulk carbon and oxygen isotope analyses of aptychi, 43 specimens in total were selected (15 from interval A, 16 from B, and 12 from C; all are isolated specimens). These specimens were pulverized and homogenized in an agate mortar. Additionally, 10 aptychi were serially sampled in order to detect ontogenetic variation in their stable isotope content (2 specimens from A, 6 from B, and 2 from C; 87 measurements in total). Carbonate samples were extracted from spot locations on the aptychi surfaces along the growth axis. Additionally, chalk matrix directly adjacent to the serially sampled aptychi was analysed, based on 8 samples.

### Microstructural observations of aptychi

Sections of aptychi were prepared and examined at the Institute of Paleobiology PAS. These sections were made perpendicular to the aptychus surface, approximately along the growth axis of the aptychus. Petrographic observations were done using a Nikon Eclipse 80i transmitted light microscope fitted with a DS-5Mc cooled camera head. Measurements were made using image analysis software (NIS Elements D software, https://www.microscope.healthcare.nikon.com/products/software/nis-elements) from digital micrographs (Nikon DSIFi2). Observations were conducted in transmitted light, which enabled quick assessment of each fossil's microstructural organization and the presence of diagenetic minerals. Selected thin sections were carbon coated and analysed using cathodoluminescence (CL) microscopy. CL analysis was conducted at the NanoFun laboratory (Institute of Paleobiology PAS), using an HC1-LM hot cathode microscope with the following parameters: electron energy 14 keV, beam current density 0.1 μAmm^−2^. For SEM studies of aptychi, selected polished sections (byproduct of cutting thin sections) were etched for 8–15 min in 8% formic acid, rinsed with Milli-Q water, and air-dried. Next, the specimens were placed on stubs with double-sided adhesive tape and sputter-coated with a conductive carbon film. Analyses were conducted at the Institute of Paleobiology PAS, using a Philips XL20 scanning electron microscope. SEM imaging provided high-resolution support for transmitted light observations; for example, SEM studies made it possible to obtain more detailed information on the preservation of the microstructure of aptychi. This instrument was operated at an acceleration voltage of 25 kV, a beam current of 98–103 nA and a spot diameter of 3.5 µm.

### Elemental analyses of aptychi

Contents of major, minor and trace elements in aptychi were examined on uncovered thin sections previously documented in transmitted light and were made using a Cameca SX-100 electron microprobe at the Joint-Institute Analytical Complex for Minerals and Synthetic Substances (Faculty of Geology, University of Warsaw, Poland). In total, 43 measurements from four aptychi were made. The mineral compositions were determined in wavelength dispersive spectral (WDS) mode on the EMPA using an accelerating potential of 15 keV, a 20 nA beam current, 1 µm beam size, peak and background counting times of 20–30 s and standard ZAF (PAP) correction procedures. A combination of natural and synthetic standards was used for calibration. The peak counting times were 10 s for major elements and 20 s for minor elements. At these durations, the average detection limits were 423 ppm for Mg; 190 ppm for Si; 232 ppm for Ca; 155 ppm for Al; 495 ppm for Sr; 517 ppm for Ba; 229 ppm for P; 189 ppm for S; 625 ppm for Fe; 593 ppm for Mn.

### Stable isotope analyses

The stable isotope composition of carbonates studied (bulk rock, foraminiferal and aptychi samples) was analysed at the Warsaw Isotope Laboratory for Dating and Environment Studies of the Polish Academy of Sciences. The samples were dissolved in 100% phosphoric acid at 70 °C, using a Kiel IV on-line carbonate preparation device, connected to a ThermoFinnigan Delta Plus mass spectrometer. The quality of the analysis was controlled by NBS-19 international standard measurements. The δ^13^C and δ^18^O values are given relative to the V-PDB standard. Analytical reproducibility was verified on the basis of the repeatability of the NBS-19 results, with an observed deviation of < 0.03‰ for δ^13^C and < 0.07‰ for δ^18^O measurements. Statistical analyses were performed using Past-free software for scientific data analysis including univariate and multivariate statistics^46^.

### Palaeotemperature calculations

Calcite temperatures were calculated using the equation of Anderson and Arthur^47^, modified by Coplen et al.^48^, assuming a δ^18^O value of − 1‰ for non-glacial sea water in accordance with palaeoposition and climate of Chełm site during the time-interval studied (compare Thibault et al^14^, p. 436):

$$\begin{aligned} {\text{T}}\;\left( {^\circ {\text{C}}} \right) & = 16{-}4.14\left( {\updelta^{18} {\text{O}}_{{\text{c}}} {-}\left( {1.03086 \, \left( {\left( {\updelta^{18} {\text{O}}_{{\text{w}}} {-}30.91} \right)/1.03091} \right) + 30.86} \right)} \right) \\ & \quad + 0.13 \, \left( {\updelta^{18} {\text{O}}_{{\text{c}}} {-}\left( {1.03086 \, \left( {\left( {\updelta^{18} {\text{O}}_{{\text{w}}} {-}30.91} \right)/1.03091} \right) + 30.86} \right)} \right)^{2} \\ \end{aligned}$$

### Sample processing in search of pyrite framboids

Samples of approximately 15 g of chalk from each of the three sampling intervals at Chełm (A–C) were studied in search of pyrite framboids. These samples were roughly grounded and then put in a solution of hydrochloric acid at a pH of 5.0 so as to dissolve the carbonate fraction. The insoluble residue was neutralized by washing it with demineralized water and ethanol. The samples were left drying for 2 days at room temperature and then roughly grounded again. The powder was placed on stubs, sputter coated with platinum and examined in a Philips XL-20 SEM (IP PAS).

### Sample processing for REE analyses

Three chalk samples from intervals A–C of the Chełm succession and a comparative sample of the upper upper Maastrichtian spiculitic limestone of opoka type from a quarry at Wola Piasecka, southeast Lublin, were studied for Rare Earth Elements (REE) content. Samples were powdered and analyzed at the Bureau Veritas Acme Labs Canada Ltd. The contents of major, minor and rare elements were analyzed using inductively coupled plasma mass spectrometry (ICP-MS) methods. The precision and accuracy of the results were better than ± 0.05% (mostly ± 0.01%) for the major elements and commonly better than ± 1 ppm for the trace elements. The concentrations of REE were normalized to Post-Archean Australian Shales (PAAS)^49^ as indicated by the subscript ‘N’. Enrichment of HREEs was calculated as Yb_N_/Nd_N_ owing to the presence of positive La anomalies and highly variable, negative Ce anomalies in shallow seawater. Ce_N_ anomalies were calculated following the relationship presented by Webb and Kamber^50^:

$${\text{Ce}}_{{\text{N}}} /{\text{Ce}}^{*}_{{\text{N}}} = {\text{Ce}}_{{\text{N}}} /\left( {0.5 \times {\text{La}}_{{\text{N}}} + 0.5 \times {\text{Pr}}_{{\text{N}}} } \right)$$

### Figure processing

All figures in this paper, including those in Supplementary Material, were prepared using CorelDraw 18 (https://www.corel.com/pl/?link=wm) and Adobe Photoshop CS3 (https://www.adobe.com/de/creativecloud/desktop-app.html?mv=affiliate&mv2=red).

## Supplementary Information

Supplementary Information.
