# Supplementary material for: Stable isotopes and predation marks shed new light on ammonoid habitat depth preferences
Source: Sci Rep. 2021 Nov 23;11:22730. doi: 10.1038/s41598-021-02236-9 (PMC8611083; doi:10.1038/s41598-021-02236-9)
Supplement: Supplementary file 1 — Supplementary Information. [file 41598_2021_2236_MOESM1_ESM.pdf]

Supplementary Information for
'Stable isotopes and predation marks shed new light on ammonoid habitat depth preferences'

Marcin Machalski, Krzysztof Owocki, Zofia Dubicka, Oksana Malchyk, Weronika Wierny

This file includes:

Geological and palaeontological background
Study section and sampling
Lithology
Stratigraphy and correlations
Macrofossil assemblages
Foraminiferal assemblages
Bathymetry
Material assessment
Taxonomy of scaphitid remains
Taphonomy of scaphitid remains
Interpretation of predation marks
Evaluation of diagenetic alteration of aptychi
Selection of foraminifera
Stable isotope data
Additional diagrams
Tables with rough isotope and palaeotemperature data
Additional research
Search for pyrite framboids
REE analyses
References
In total 19 **SI Figures** and 13 **SI Tables**.

Geological and palaeontological background

Study section and sampling. The large working chalk quarry of the cement company *Cemex Polska* is situated in the eastern part of the town of Chełm, eastern Poland (SI-Fig. 1). Currently, the chalk is accessible along four exploitation levels that are referred to, from the top to the bottom, as II, III, IV, and V; the former level I has been completely excavated. A portion of the section below level V was temporarily exposed in a dewatering trench and designated level VI by Dubicka and Peryt (2011). A total thickness of the chalk sequence currently exposed at Chełm is c. 40 m, with the top of the working level II located at 203 m a.s.l. (above sea level), and the bottom of level VI at 164 m a.s.l. (Dubicka and Peryt, 2011, fig. 2; see Fig. 2, SI-Fig. 2).

The chalk at Chełm is macroscopically homogeneous and because any lithological marker horizons are lacking, the working levels are the sole reference for sampling (Dubicka and Peryt, 2011). The materials studied were recovered in two ways (Fig. 2; SI-Fig. 2):

1) Sampling of the entire section in regular intervals from the bottom to the top (Fig. 2; SI-Fig. 2). The sample numbers are composed of Roman numerals to indicate exploitation levels, and Arabic numerals for sample numbers within any of the sampled levels (see also fig. 2 in Dubicka and Peryt, 2011). These samples were collected approximately every 1 m along the quarry face, except for some intervals covered by scree. The samples provided data for foraminiferal and isotope profiles (Fig. 2; SI-Fig. 2).

2) Sampling of three narrow chalk intervals A–C, each 2 m thick (Fig. 2, SI-Fig. 1). Due to the way in which the chalk is excavated at the quarry, only these intervals made it possible to collect macrofossil specimens and samples that were sufficient for the present study in terms of their quality and quantity. The chalk is mined by excavators that move along the quarry walls. During these operations, almost all thickness of the chalk is removed from the quarry, except for the bottom two metres, which are left at the floor of each level as elongated heaps of chalk, which parallel the quarry faces (SI-Fig. 1B,C). If such a 'leftover' remains on the quarry floor long enough (several months at least), the chalk disintegrates, reaching a stage where it is suitable for effective macro-fossil collecting (except for conspicuous objects, such as pyritised echinoid tests, macrofossils are not easily spotted on fresh chalk surfaces). Our sampling intervals A, B, and C were situated at the bottom portions of the levels V, IV, and III, respectively. The sampling intervals A, B, C correspond to the intervals of 166–168, 176–178, and 186–188 m above mean sea level, respectively (Fig. 2, SI-Fig. 2).

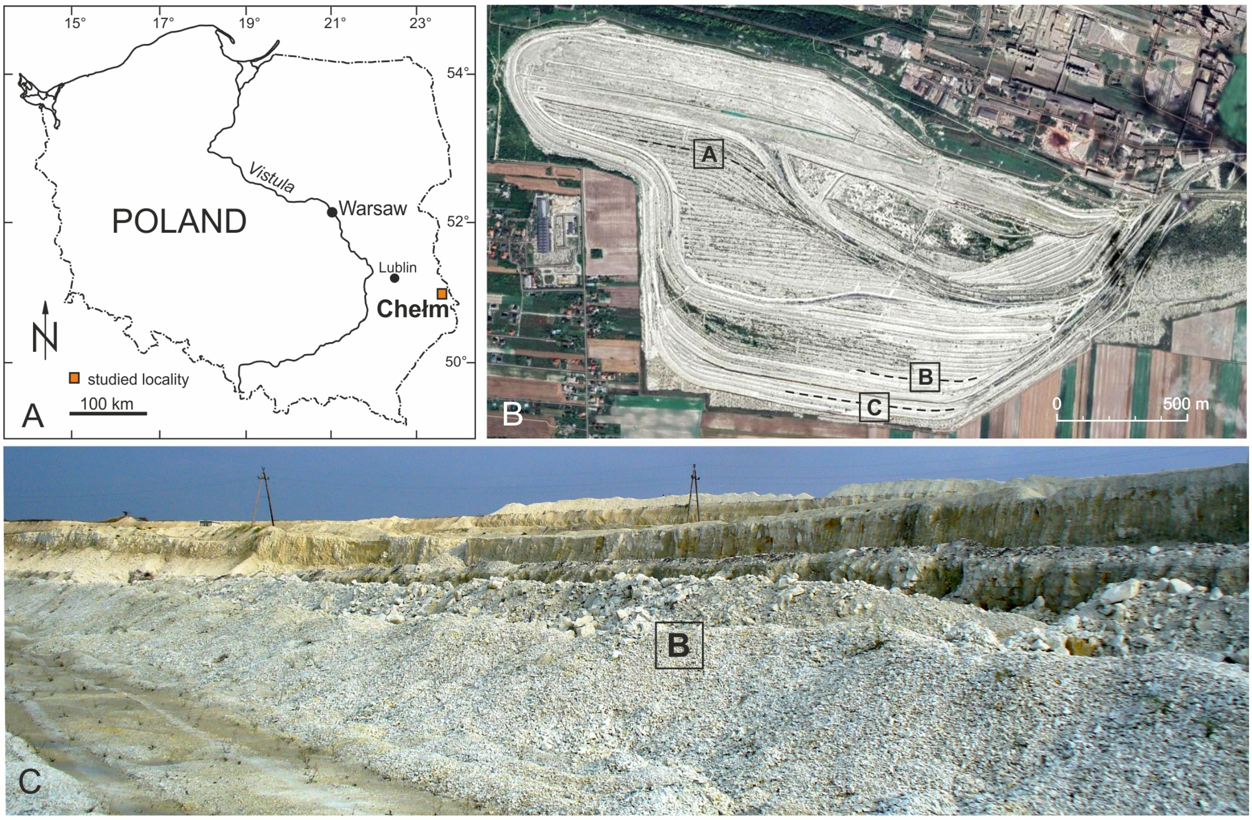

SI Figure 1. The locality studied. (**A**) Location of the Chełm site in Poland (redrawn with modification from Machalski, 2021, fig. 1). (**B**) Satellite view of the Chełm chalk pit (Google Maps, https://www.google.com/maps/place/Chełm) with position of sampling intervals A–C indicated. Parallel strips on the quarry floor are successive elongated heaps of the chalk, from which the macro- and microfossil material may be collected. (**C**) Close-up view of two elongated heaps of disintegrated chalk from sampling interval B; all fossils and samples from interval B studied herein come from such 'leftovers'.

Lithology. The chalk exposed at Chełm is a fine-grained carbonate deposit composed almost exclusively of coccoliths. At levels VI to IV, the chalk is light-grey in color when fresh, due to a slightly higher admixture of clay. Higher up, at levels III and II, fresh chalk is creamy in color due to a decrease in clay content. The chalk at all levels becomes almost white when dry and relatively easily disintegrates in open air.

Flint layers, burrowed omission surfaces, bored and/or mineralized hardgrounds and other marker beds, which are so characteristic of many white chalk sites in Europe (Surlyk and Birkelund, 1977; Surlyk et al., 2003; Hansen and Surlyk, 2014; Wilmsen and Niebuhr, 2017; Bojanowski et al., 2017) are absent at Chełm. The only indications that the chalk strata lie almost horizontally are provided by the orientation of some fossils, for instance echinoid tests buried in life position (Dubicka and Peryt, 2011).

The chalk appears totally bioturbated throughout the section. Occassionally, deep-tier burrows (*Chondrites* and *Zoophycos*) are discernible on fresh surfaces of chalk at the lower levels of the quarry, being accentuated by slight differences in colour between their infills and surrounding sediment. Higher up, the individual burrows are not recognisable, which reflect a decreased clay content (a higher content of clay enhances the visibility of traces in the European chalk, see Lauridsen et al., 2011).

The chalk exposed at levels VI to IV contains abundant pyrite developed as grains, nodules and fossil impregnations. Relatively large, heavily pyritized echinoid tests, sponge fragments and burrow infills are noted at these levels. In contrast, the chalk exposed at levels III and II is devoid of pyrite. At Chełm, we failed to find pyrite framboids that Tagliavento et al. (2020) recorded from Maastrichtian marly chalk in Denmark.

Stratigraphy and correlations. According to Machalski (2005) and Dubicka and Peryt (2011), the chalk succession at Chełm corresponds to the lower upper Maastrichtian *Belemnitella junior* and *Belemnitella junior-Spyridoceramus tegulatus* zones in the standard subdivisions of the Boreal Maastrichtian in Europe (e.g., Schulz and Schmid, 1983). In ammonoid terms, the whole section was previously assigned to the *Hoploscaphites constrictus lvivensis* Zone (Machalski, 2012; Dubicka and Peryt, 2011; Walaszczyk et al. 2016), based on the occurrence of the eponymous subspecies (Machalski, 2005), but the current data allow us to assign only the lower portion of the section (sampling intervals A and B) to this zone.

The planktic foraminifer assemblages recovered from Chełm are characterized by a considerable number of representatives of *Globigerinelloides* and *Heterohelix* and the presence of *Guembelitria cretacea.* This indicates the lowest part of the *Guembelitria cretacea* Zone *sensu* Peryt (1980). Dubicka and Peryt (2012) assigned the Chełm section to their local foraminiferal zones VIII (lowest part), IX (bulk of section), and X (top of section). This interval also belongs to the lowermost part of the so-called zone without *Stensioeina* (Witwicka, 1958).

The above-mentioned biostratigraphic assignments can be refined by correlation of the Chełm section with the Stevns-1 core section in Denmark, which is a reference section for the Maastrichtian of the Boreal Chalk Sea (Boussaha et al., 2012; Thibault et al., 2012; Surlyk et al., 2013). Our correlation is based primarily on planktic foraminiferal data, and is additionally supported by stable carbon isotope data (SI-Fig. 2). Two planktic foraminiferal events, recognized in both sections, seem to be of key importance here. These are: (1) a temporary disappearance of deeper-dwelling planktic foraminifera and (2) a rapid burst in abundance of shallow-water heterohelicids (SI-Fig. 2). For the first event, we wish to indicate that this is expressed by the disappearance of *Rugoglobigerina* in the Stevns-1 core, while at Chełm it is

characterized by the last occurrence of globotruncanids. This may be related to differences between two distinct Cretaceous planktic foraminiferal bioprovinces – the Chełm site is located in the Transitional Bioprovince, whereas the Stevns-1 section belongs to the Boreal Bioprovince (Schneibnerovà, 1971). The foraminiferal correlation is consistent with the shape of the carbon isotope curve in both sections, which display a deflection toward lower values near the heterohelicid burst (SI-Fig. 2).

Based on the above, we propose that the Chełm section corresponds to the interval between c. 180 and 115 m at Stevns-1 (SI-Fig. 2). The latter interval represents the Hvidskud Member of the Møns Klint Formation, which comprises the topmost part of UC19, the entire UC20a and the lower part of UC20b-c nannofossil zones, and three magnetostratigraphic zones: 31n, 30r and 30n. In terms of the 405 kyr Maastrichtian astronomical cycles (Surlyk et al., 2013), the Chełm section corresponds to the interval from the upper part of Ma9 to the middle part of Ma5 as distinguished in the Stevns-1 core. This means a duration of more than one million years for the Chełm succession. By extrapolation, our sampling intervals A, B, and C (Fig. 2, SI-Fig. 2) may be approximately dated as 69.0, 68.7, and 68.3 Ma, respectively.

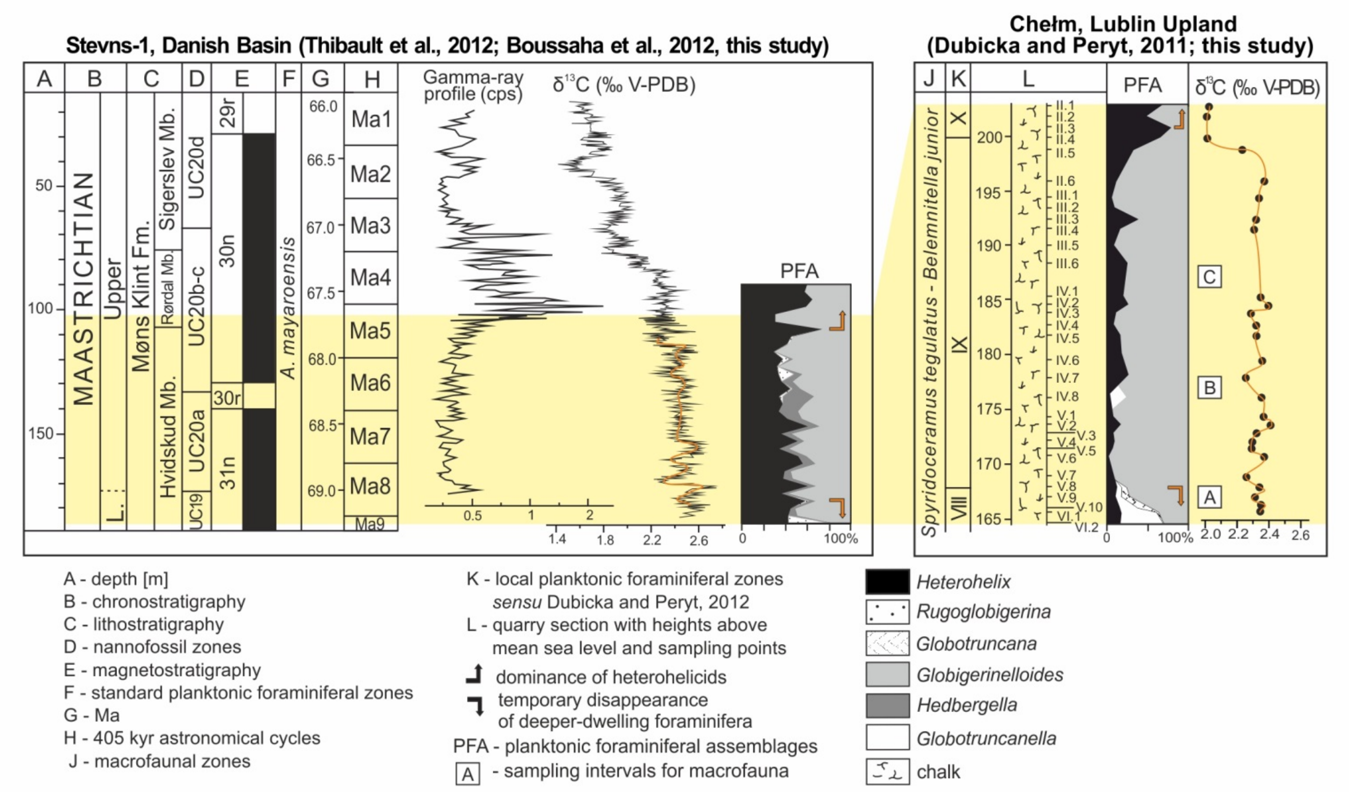

SI Figure 2. Correlation (in yellow) between the Stevns-1 reference core in Denmark and the Chełm succession. Samples from Stevns-1 provided by Nicholas Thibault (University of Copenhagen).

Macrofossil assemblages. As noted earlier, the macrofossil material studied was collected from intervals A–C in the quarry. In general, the Chełm chalk contains macrofossil assemblages of rather poor diversity and low abundance.

The nekton is dominated by ammonoids, the moulds of which are not uncommon throughout the section. These include fragmentarily preserved *Baculites* spp. and the scaphitids *Hoploscaphites constrictus lvivensis* (intervals A and B) and *H. c.* aff. *crassus* (interval C). Extremely rare individuals of other ammonoids, including *Glyptoxoceras* sp., and the scaphitids *Hoploscaphites felderi*, *H. tenuistriatus*, *H. schmidi*, and *Acanthoscaphites varians blaszkiewiczi*

have been also recorded (Machalski, 2005; Machalski et al., 2008). Aptychi of *Hoploscaphites constrictus* (see Material Assessment) are relatively common throughout the section; a few aptychi attributable to *Baculites* have been identified as well. Nautilids are represented by rare individuals of *Eutrephoceras aquisgranense*, *E. dekayi*, *E.* sp. and *Cymatoceras intrasiphonatum* (Malchyk, unpublished). Coleoid cephalopods are represented by extremely rare rostra of belemnites. Dubicka and Peryt (2011) reported *Belemnitella junior* (three specimens, quarry levels VI, V and III), and *B. lwowensis* (one specimen, level V). Notably, all these specimens represent subadult to adult growth stages. This suggests that the belemnite guards from Chełm belonged to stranded individuals buried far from their optimum, shallow-water life habitats (Christensen, 1976; Surlyk and Birkelund, 1977). Remains of nektic invertebrates (bony fish scales, shark teeth) are rare; a fragmentary, partially articulated skeleton of a bony fish was recovered from interval C.

Benthos is represented by an assemblage of rather low density and richness, dominated by bivalves and brachiopods. *Spyridoceramus tegulatus* is the sole representative of inoceramids, and is relatively common throughout the entire section (compare Dubicka and Peryt, 2011). Other bivalves represented are *Oxytoma danica*, *Phelopteria pectenoidea*, *Pycnodonte vesicularis*, *Spondylus dutempleanus*, *Syncyclonema*? *semiplicata*, *Limea* sp., *Limatula* sp., and *Atreta nilssoni.* All these taxa are epifaunal suspension-feeders with calcitic shells. Moulds of the originally aragonitic *Nucula* and *Nuculana*, which are infaunal deposit-feeders, are not uncommon either. All these bivalves seem to be distributed in equal numbers between the sampling intervals, with exception of *Atreta nilssoni* and cementing juveniles of *Pycnodonte vesicularis*, which are more abundant as encrustations on ammonoid conchs in interval C. Occasionally, inoceramids and other pteriomorphs form monospecific clusters, which in part my reflect the pseudoplanktic mode of life of these bysally attached bivalves (compare Kauffman, 1981; Wignall and Simms, 1990). Rare moulds of small gastropods, mainly *Aporrhais* sp. and *Turritella* sp., occur in all intervals. Echinoids are relatively common, predominated by tests of semi-infaunal mobile deposit-feeding holasteroids (*Echinocorys* ex gr. *limburgica*/*duponti*) as well as primary spines of cidaroids, which represented mobile epifaunal omnivores (taxonomic identifications by John W.M. Jagt, unpublished). As noted by Dubicka and Peryt (2011), *Echinocorys* tests are abundant in intervals A and B, and rare in C. Brachiopods are represented by large terebratulids, *Carneithyris* sp. and *Neoliothyrina obesa*, and a rhynchonellid, *Cretirhynchia* sp. Additionally, a rather poor assemblage of micromorphic brachiopods, dominated by long-ranging species, was recovered by washing the sediment from all levels of the quarry (Bitner, 2008). The life habits of these brachiopods varied; the larger forms represent epifaunal suspension-feeders (Surlyk, 1972). Rare indeterminate siliceous sponges, solitary corals, isolated cirripede plates, encrusting bryozoans and serpulid tubes have also been collected.

In terms of taphonomy, the preservation of macrofossil assemblages from Chełm is typical of the European white chalk (e.g., Nestler, 1965; Reich and Frenzel, 2002; Hansen and Surlyk, 2014). Specifically, there are no fossil concentrations (pavements, stringers) at Chełm which might be ascribed to storms or strong bottom-currents. Such accumulations occur in the stratigraphically younger Maastrichtian opoka facies of central and eastern Poland, which was usually laid down at shallower depths than the chalk (Machalski and Malchyk, 2019).

The body fossil record of benthos at Chełm is supplemented by ichnofossils, which may be observed in the field (see above) and on polished slabs of chalk after enhancement of their visibility by oil painting (Bushinsky method, see Bromley, 1981). This latter procedure provided, however, satisfactory results only in the case of samples from interval A. These slabs reveal a suite of trace fosssils, including deeper-tier *Chondrites* (large and small) and *Zoophycos*, superimposed on less distinctive shallower-tier traces, including *Taenidium*, *Planolites* and

Thalassinoides, and a mottled background ichnofabric (see Ekdale and Bromley, 1991). This is a typical white chalk ichnofacies, pointing to relatively deep water offshore conditions (Ekdale and Bromley, 1984; Surlyk et al., 2003). Slabs from intervals B and C failed to provide conclusive ichnological data in view of poor visibility of traces, except for some *Taenidium* and *Chondrites*, and the obliteration of the slab surfaces by multiple generations of subsolution seams.

Foraminiferal assemblages. The foraminiferal record of the Chełm section was discussed in detail by Dubicka and Peryt (2011, 2012). In brief, the benthic foraminifera are similar in the entire section and are of high diversity comprising over thirty genera (Dubicka and Peryt, 2011), representing epifaunal (e.g., *Gavelinella*, *Cibicidoides*, *Anomalinoides*), shallow-infaunal (e.g., *Gyroidinoides*, *Valvulineria*, *Ataxophragmium*) and deep-infaunal (e.g., *Tritaxia*, *Arenobulimina*, *Praebulimina*, *Coryphostoma*) forms. Both calcareous and agglutinated benthic foraminifera are present, with a high percentage of agglutinated forms (30% of the entire assemblage) and infaunal forms (30-50%; see Dubicka and Peryt, 2012, fig. 8). The benthic foraminifera assemblage from Chełm indicates oxic bottom conditions throughout the section (Dubicka and Peryt, 2011).

The planktic foraminifera are more changeable within the section (SI-Fig. 2; see also Dubicka and Peryt, 2011, fig. 11; Dubicka and Peryt, 2012, fig. 2). In general, they are dominated by representatives of *Globigerinelloides*, followed by *Heterohelix.* The abundance of *Heterohelix* increases at level II and in the uppermost part of the section these biserial planktic foraminifera prevail over *Globigerinelloides* (Dubicka and Peryt, 2011). At levels VI and V, representatives of *Globotruncanella* and *Globotruncana* are common, declining in abundance up to their disappearance upward of level V (SI-Fig. 2). Following Leckie (1987) and Dubicka and Peryt (2012), *Heterohelix*, *Globigerinelloides*, *Globotruncanella*, and *Globotruncana* inhabited progressively larger depths (with *Heterohelix* representing the shallowest, and *Globotruncana* the deepest habitats).

Bathymetry. In general, the Chełm chalk matches the characteristics of the benthos-poor chalk in the Boreal Chalk Sea facies model (Surlyk et al., 2003, fig. 13.7). The benthos-poor chalk represents the deepest epicontinental chalk facies, deposited below the photic zone and storm-wave base (Surlyk et al., 2003). Like other examples of this facies, the Chełm chalk is monotonous, lacks hardgrounds, omission surfaces, scour horizons, tabular fossil concentrations, and flint nodules. Its macrofossil content is typified by a rather poor macrobenthic assemblage, a predominance of *Zoophycos* burrows (at least in the lower levels), a relative abundance of ammonoids and extreme rarity of belemnites which are represented by adult individuals only (compare Surlyk and Birkelund, 1977; Surlyk et al., 2003; Hansen and Surlyk, 2014).

According to Håkansson et al. (1974), the Maastrichtian chalk of Europe was deposited at depths ranging down to 250 m. More precise bathymetric estimates for the Chełm succession may be gained from planktic foraminiferal data (Dubicka and Peryt, 2011, 2012). The lowermost part of the section (level VI and lower part of V) yielded deepest-water dwelling foraminifera such as *Globotruncana*, *Contusotruncana* and *Globotruncanella*, which completely disappear upsection (SI-Fig. 2). The bulk of the section (from high in level V to low in level II) is punctuated by subtle variations in the planktic foraminiferal spectra, which are dominated by shallowest-water *Heterohelix* and deeper-water *Globigerinelloides*. Nonetheless, there is a general trend toward an upward increase in abundance of the shallowest-dwelling small biserial *Heterohelix* in this succession. *Heterohelix* bursts rapidly in numbers at level II. Such distribution of planktic foraminifera indicates a progressive shallowing of the sea during

deposition (Dubicka and Peryt, 2012). According to these authors, this shallowing trend is a regional record of a marine regression following the mid-Maastrichtan global sea level rise, identified by Hancock (1993) as Peak 4. Application of depth ranges inferred for the Cretaceous planktic foraminifera (Leckie, 1987) allows us to propose that the lowermost portion of the Chełm section was deposited at a depth below 100 m. Its higher portions were deposited at progressively shallower depths, with the shallowest environment, possibly around several dozens of meters, coinciding with level II. Specifically, sampling interval A is located within the range of the deepest-dwelling foraminifera, and interval C coincides with the level with relatively abundant small heterohelicids, and no deeper-water planktic foraminifera at all (SI-Fig. 2). Therefore, we interpret the chalk from interval A to have been deposited at a depth of around 100 m and that from C at a much shallower depth, probably several dozen meters only. Chalk exposed in interval B seems to have been laid down at an intermediate depth, based on the extrapolation from the overall trend. This is supported by a minor incursion of deeper-water *Globotruncanella* near the base of this interval (sample IV. 8 in SI-Fig. 2). Results of Rare Earth Elements (REE) analyses confirm the bathymetric interpretations based on foraminifera (see below).

Material assessment

Taxonomy of scaphitid remains

The mould-based taxa. For explanations of morphological terms for shell and aptychus of *Hoploscaphites constrictus* (J. Sowerby, 1817), the reader is referred to Machalski (2021, fig. 2). Of this species, two morphs are known which conventionally have been interpreted as sexual dimorphs (Makowski, 1962; Machalski, 2005). Small specimens with some distinctive morphological features (microconchs) are presumed to be males, whereas the larger individuals (macroconchs) are conventionally assumed to represent females (SI-Fig. 3).

In total, 187 moulds of *H. constrictus* were collected at Chełm for this project. Amongst these, 24 macroconchs are from interval A, 71 macroconchs and 1 microconch from B, and 89 macroconchs and 2 microconchs from C. Therefore, there are only three microconch individuals in the present material. Birkelund (1982) recorded similar macroconch vs. microconch proportions (260 vs. 1) from the Maastrichtian chalk exposed at Hemmoor, NW Germany.

Hoploscaphites constrictus forms a monospecific evolutionary lineage which ranges from the earliest Maastrichtian to the earliest Danian, with some short-term survivors reported from the Danian Cerithium Limestone Member of the Stevns Klint section (Machalski and Heinberg, 2005; Machalski, 2005; Landman et al., 2014). The late Maastrichtian portion of this lineage was subdivided into three successive temporal subspecies (Machalski, 2005). These are, in ascending order, *Hoploscaphites constrictus lvivensis* Machalski, 2005, *H. c. crassus* (Łopuski, 1911), and *H. c. johnjagti* Machalski, 2005 (SI-Fig. 3). Typical specimens of the first two subspecies are illustrated in SI-Fig. 4. This subdivision of the *H. constrictus* lineage has been used for the biostratigraphic subdivision of the upper Maastrichtian in Poland (Machalski, 2012; Dubicka and Peryt, 2011, 2012; Walaszczyk et al., 2016).

Machalski (2005) defined *Hoploscaphites constrictus lvivensis* Machalski, 2005 on the basis of material from Chełm, using a populational approach to the species concept. The holotype of this subspecies is ZPAL Am. 12/1051 (fig. 5C in Machalski, 2005; refigured here in Figs. 1a, SI-Fig. 4A–D). The diagnostic feature of *H. c. lvivensis* is an adapertural decrease in distance between ventrolateral tubercles in adult macroconchs (Machalski, 2005). Machalski also

redefined *H. c. crassus* (Łopuski, 1911) by the absence of ribbing on a large sector of body chamber in adult macroconchs (Machalski, 2005). Amongst the present specimens of *H. constrictus* from Chełm, those from sampling intervals A and B conform the definition of *H. v. lvivensis* (SI-Fig. 5E–I). However, specimens from interval C (SI-Fig. 5A–D) typically display a slightly different morphology in that the distance between the ventrolateral tubercles does not decrease towards the aperture. Moreover, the tubercles usually do not reach the aperture as in typical *H. c. lvivensis*, being instead replaced in the final sector of the hook by a zone of fine ribbing. This places these specimens closer to the chronologically younger *H. c. crassus*. On the other hand, flanks of the body chamber in most of specimens from interval C at Chełm are covered with wide and distant ribs, unlike typical *H. c. crassus*. In view of the above differences, individuals from interval C at Chełm are here provisionally referred to as *H. c.* aff. *crassus*.

Machalski (2005) based description and definition of *H. c. lvivensis* on a collection of 227 specimens from Chełm, mostly macroconchs. These fossils were collected on heaps of disintegrated chalk near the entrance to the quarry. According to the information provided by the quarry staff, these heaps originated from levels IV and III (that is, from intervals B and C). However, after examination of the new material collected *in situ*, it is clear that the material of Machalski (2005) originated from levels V and IV, and not from III (from intervals A and B and not C). This error concerns the holotype of *H. c. lvivensis* ZPAL Am. 12/1051 and a specimen with double-valved aptychus illustrated by Machalski (2005, fig. 26A1, refigured in Fig. 1b). Both finds are not from level III as stated by Machalski (2005), but from intervals A or B.

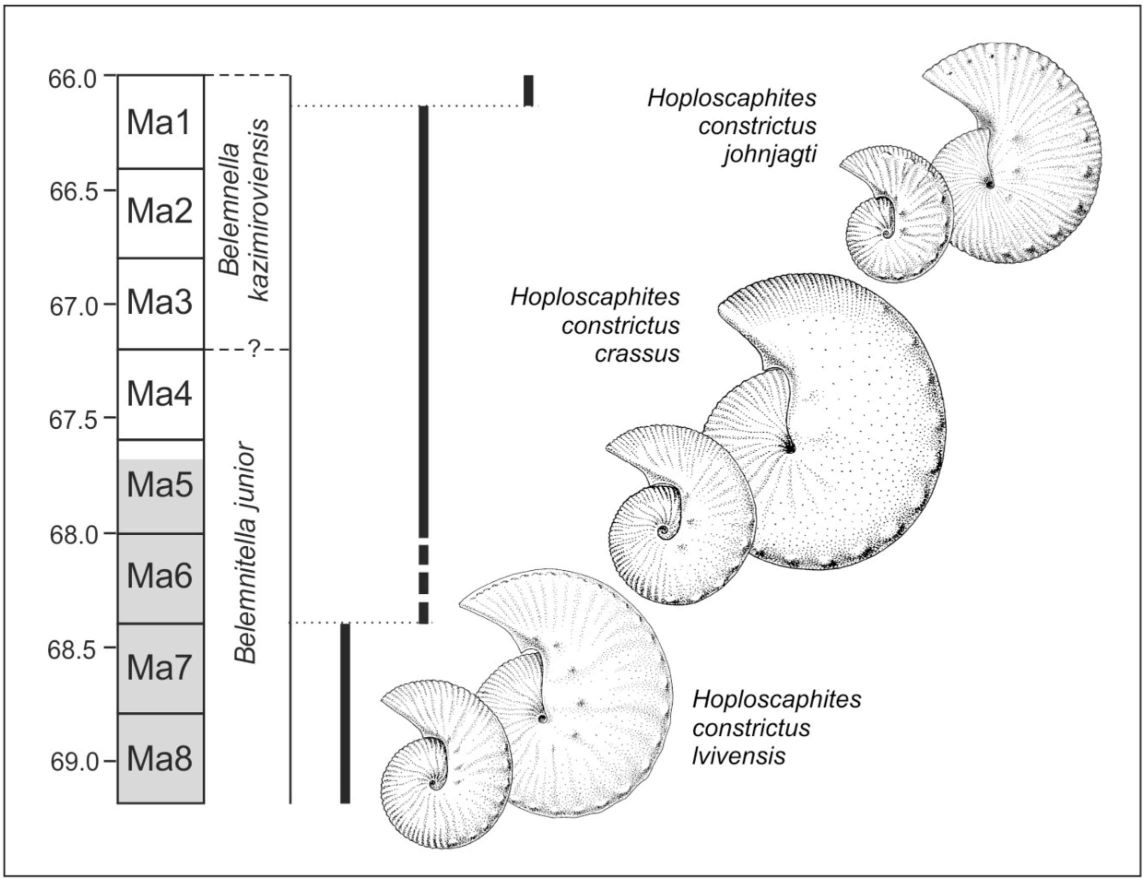

SI Figure 3. Evolution and stratigraphic ranges of three successive subspecies of *Hoploscaphites constrictus* during the late Maastrichtian; each pair of specimens is composed of a microconch (left) and macroconch (right). Modified from Machalski (2005, fig. 8). Correlation with Maastrichtian astronomical

cycles Ma8-Ma1 is tentative, based partially on this work and partially on the astronomical calibration of the ammonoid-bearing sections from the Maastrichtian type area (Keutgen, 2018, fig. 13). The grey interval corresponds to the Chełm succession (SI-Fig. 2). Tentative range of *H. c.* aff. *crassus*, intermediate between *H. c. lvivensis* and *H. c. crassus*, is marked with dashed line.

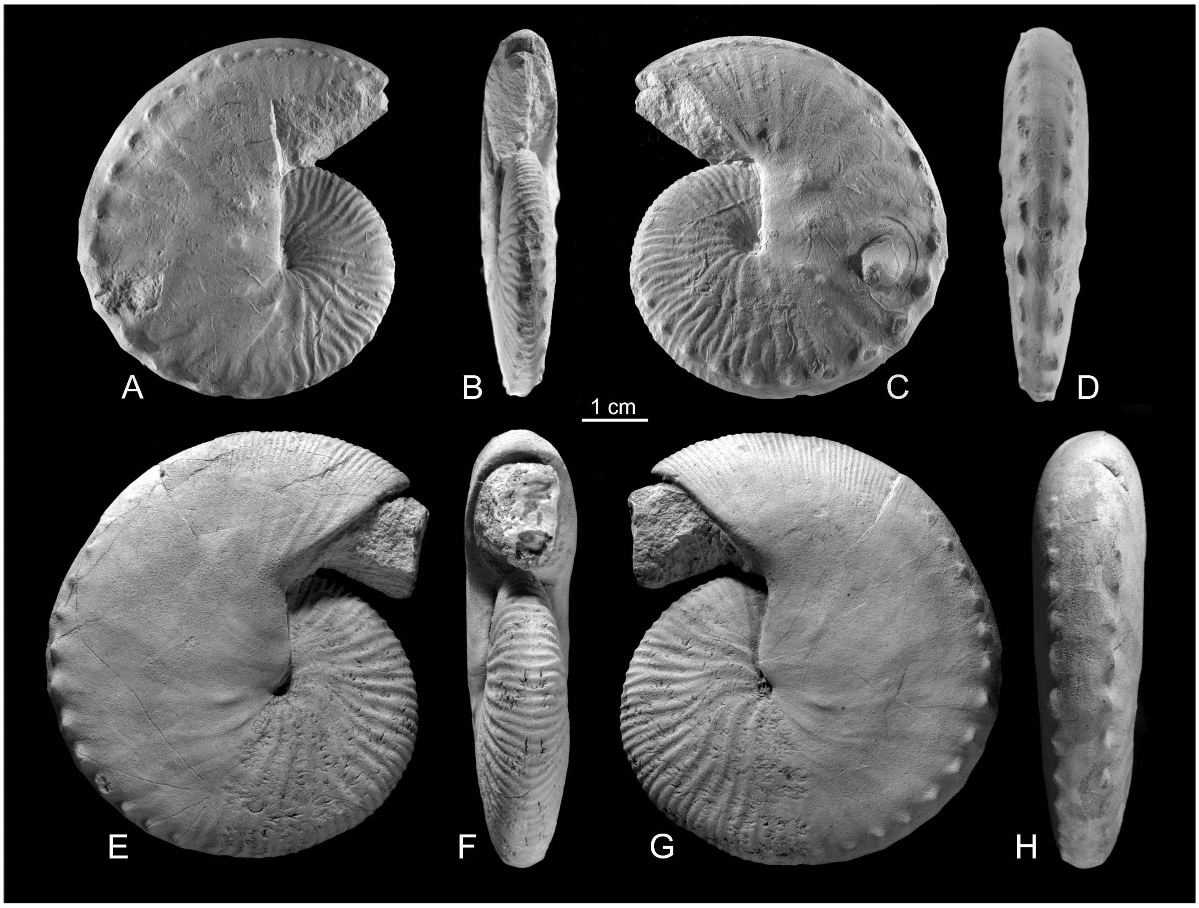

SI Figure 4. (**A–D**) *Hoploscaphites constrictus lvivensis* Machalski, 2005, holotype ZPAL Am. 12/1051, Chełm, interval A or B. (**E–H**) A typical specimen of *H. c. crassus* (Łopuski, 1911), specimen ZPAL Am. 12/62, Nasiłów.

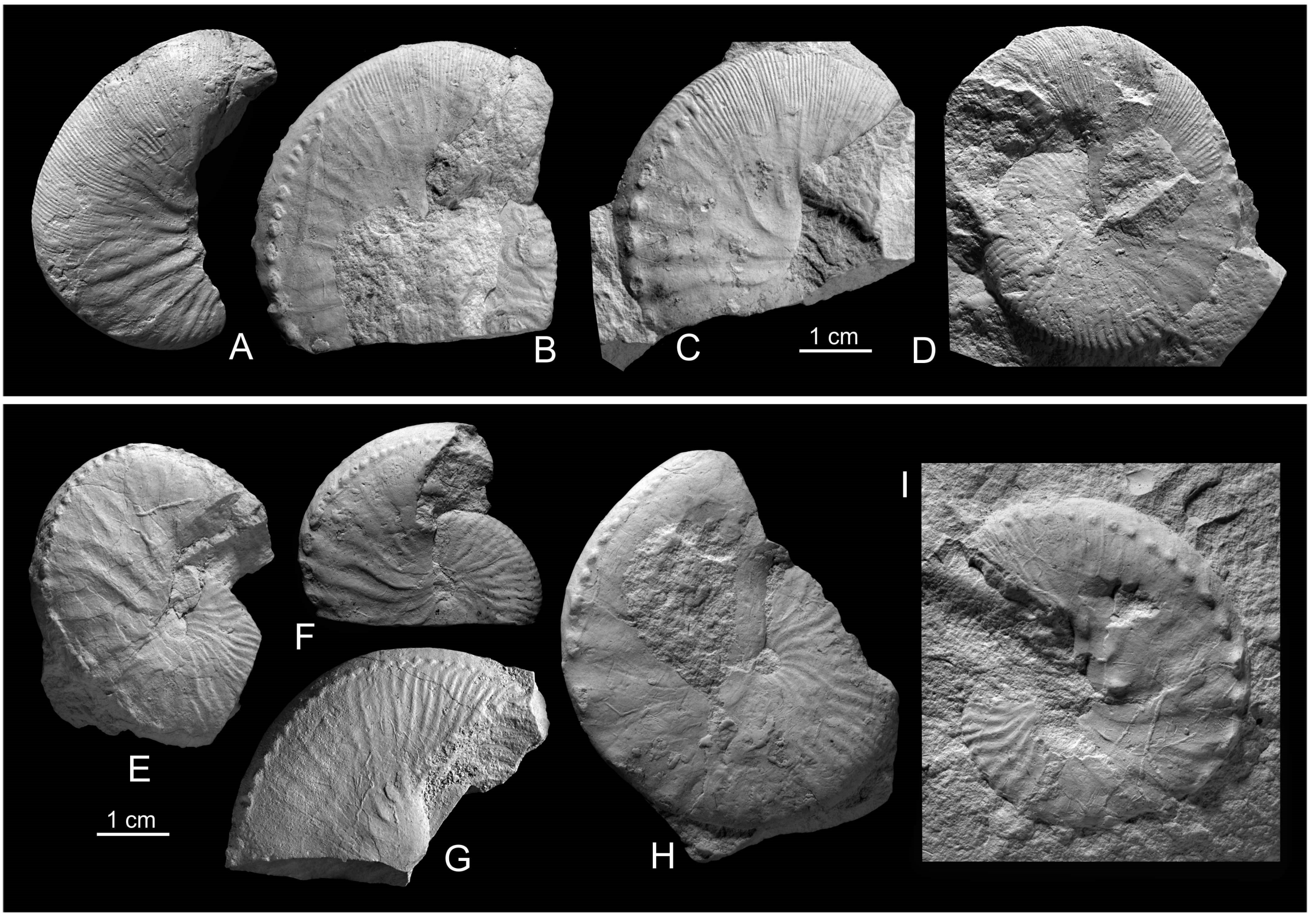

SI Figure 5. (**A–D**) *Hoploscaphites constrictus* aff. *crassus*; (A) ZPAL Am. 12/1382; (B) ZPAL Am. 12/ 1376; (C) ZPAL Am. 12/1377; (D) ZPAL Am. 12/1379. All specimens from interval C at Chełm. (**E–I**) *H. c. lvivensis* Machalski, 2005; (E) ZPAL Am. 12/ 1390; (F) ZPAL Am. 12/1389; (G) ZPAL Am. 12/1393; (H) ZPAL Am. 12/1387; (I) ZPAL Am. 12/1392. Specimen F is from interval A, specimens E, G-I from interval B at Chełm. Specimens in D and H bear lateral predation marks.

Aptychi. These are paired calcitic outer plates of the chitinous ammonoid lower jaws (see Kruta and Landman, 2008; Landman et al., 2012; Tanabe et al. 2015). The term aptychus is used for a pair of plates and single plate as well (Machalski, 2021). As far as the aptychi from Chełm are concerned (Fig. 1c, SI-Figs. 6, 7), they are represented by 130 isolated specimens of which 52 are preserved in pairs. Additionally, four aptychi were found in interval C in association with moulds of *H. constrictus*, either inside body chambers or in a close proximity (SI-Fig. 7). The isolated aptychi belong to the same morphotype as the aptychi found associated with the moulds of *H. constrictus*. Aptychi of this type are generally attributed to scaphitids (non-Linnean form genus 'Striaptychus' of Trauth, 1928; see e.g., Parent et al., 2014; Machalski, 2021). Baculitid aptychi, which occur as rarities at Chełm, are morphologically sufficiently distinctive so as not to be confused with those of scaphitids (see e.g., Larson and Landman, 2017). Other scaphitids at Chełm are extremely rare and some of them attained much greater shell sizes than *H. constrictus* (e.g., *Acanthoscaphites* and *H. schmidi*, see Machalski, 2005). Microconchs of *H. constrictus* are extremely rare as well (see above). In view of the above data, the scaphitid aptychi from sampling intervals A and B may be safely interpreted as belonging to macroconch individuals of *H. c. lvivensis* and those from interval C to macroconchs of *H. c.* aff. *crassus*.

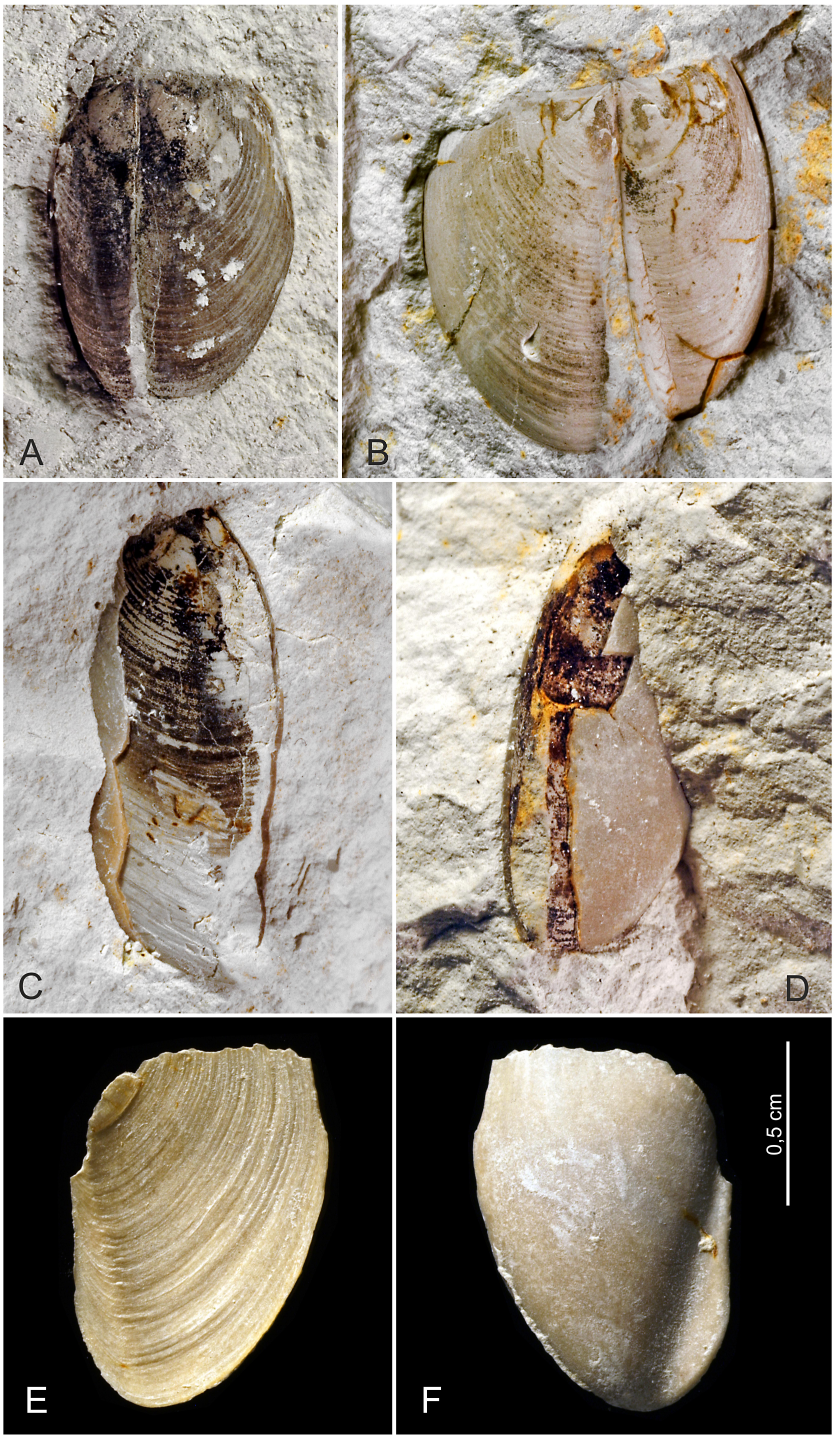

SI Figure 6. Aptychi attributable to *Hoploscaphites constrictus* from Chełm. (**A**) ZPAL Am. 12/547, a pair of aptychi in ventral view, preserved as convex-up internal moulds with carbonized remnants of the originally chitinous jaw and growth increments reproducing ridges and grooves on the dorsal surfaces of the original aptychi, interval A or B. (**B**) ZPAL Am. 24/113, a pair of aptychi in ventral view, preserved in 'butterfly' position as convex-up internal moulds with growth increments, remnants of the original calcitic aptychus are visible to the right, interval A. (**C**) ZPAL Am. 24/116, a pair of aptychi in 'folio' position, ventral view; remnants of the original calcitic aptychus are visible to the left, the remainder of the specimen is preserved as internal mould, interval B. (**D**) ZPAL Am. 24/115, a pair of aptychi with original calcitic material in 'folio' position, interval B. (**E–F**) ZPAL Am. 24/104, an isolated aptychus in dorsal (E) and ventral (F) views, interval C.

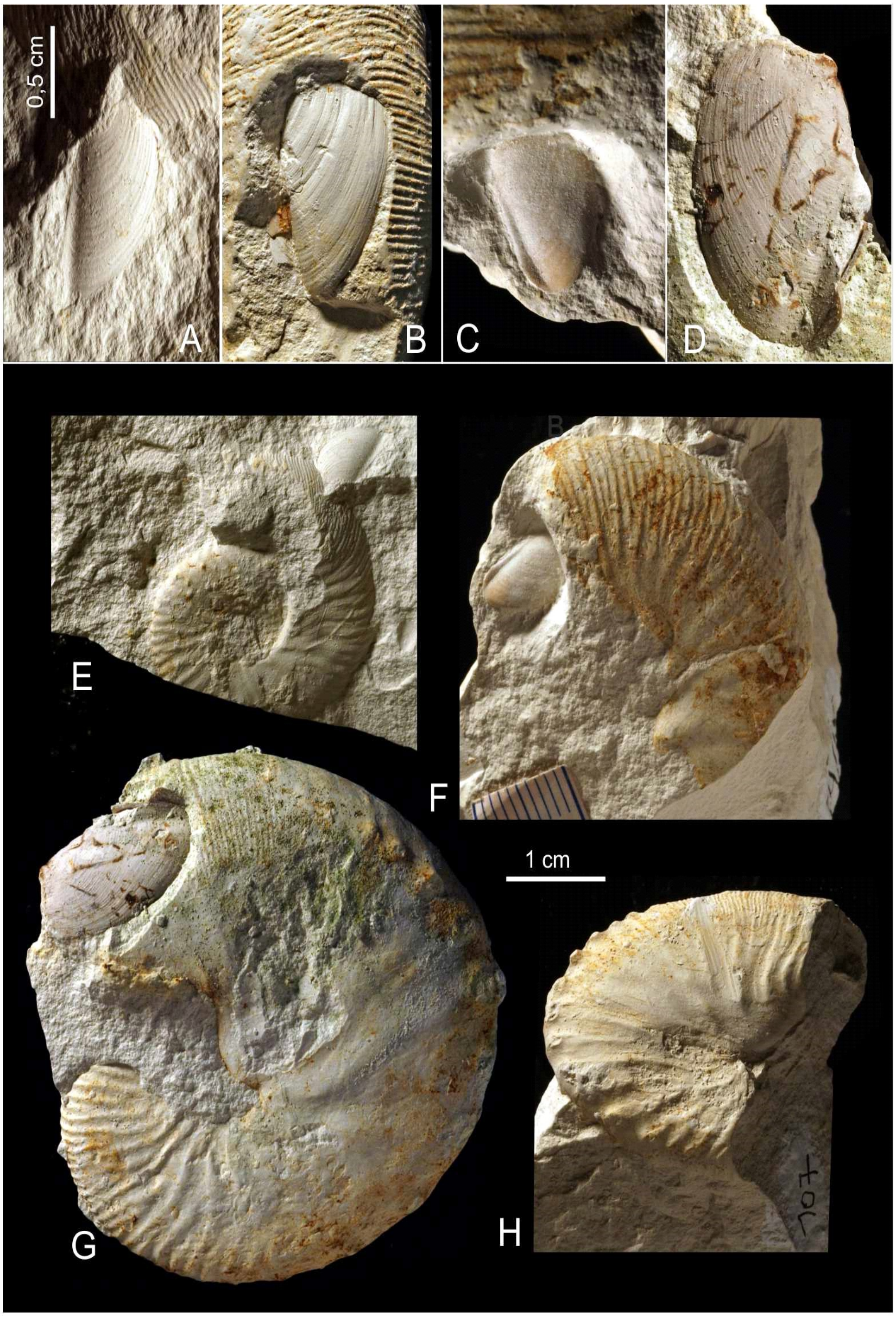

SI Figure 7. Aptychi in close association with moulds of *Hoploscaphites constrictus* aff. *crassus* from interval C at Chełm. (**A, E**) ZPAL Am. 24/108. (**B, H**) ZPAL Am. 24/107. (**C, F**) ZPAL Am. 24/106. (**D, G**) ZPAL Am. 24/105. Mould-specimens in F, G, H are macroconchs, specimen in E is a microconch. Among aptychi, specimens in A and B are internal moulds of isolated aptychi with replicas of the dorsal surfaces of the original aptychus; specimen in C is the aptychus in calcitic preservation in ventral view; specimen in D is the internal mould of the right valve of the double-valved specimen in 'folio' position, in ventral view.

Taphonomy of scaphitid remains

At Chełm specimens of *Hoploscaphites constrictus* are preserved as moulds (steinkerns) due to diagenetic dissolution of the originally aragonitic shells of these cephalopods, a phenomenon typical of white chalk and other permeable carbonates in the Boreal Chalk Sea of Europe (Janiszewska et al., 2018). Typically, the moulds occur scattered in the sediment, although in some places fossils of *Hoploscaphites*, both moulds and isolated aptychi, are much more abundant than in others. These concentrations do not reveal any size-sorting or crushing of specimens which coud be attributed to the activity of hydromechanical agents. Therefore, they are best interpreted as reflecting a gregarious mode of life of scaphitid ammonoids (compare Landman et al., 2007).

Preservation of aptychi also provides important clues for understanding the nature of the Chełm fossil assemblages. As pointed out above, most of the aptychi are found in isolation from the shells, but almost half of these have been recognized as double-valved specimens. This is a minimum estimate as in many cases it was impossible to recognize whether an aptychus found on a piece of chalk originally belonged to a double-valved specimen or not. The bivalved aptychi are preserved in a splayed-off ('butterfly') position (SI-Fig. 6A,B) or in a closed ('folio') position (SI-Fig. 6C,D, compare Seilacher, 1993).

Preservation of articulated double-valved aptychi indicates that the underlying chitinous material of the lower jaw was still present at the moment of burial of these specimens. In fact, carbonaceous remnants of the lower jaw are still present in many specimens recovered from intervals A and B (SI-Fig. 6A,C,D). In contrast, none of the aptychi from interval C preserves such remnants. The organic ammonoid jaws have generally a much lower preservational potential than calcareous aptychi, being usually reported from early-diagenetic carbonate nodules or laminated stagnant deposits (Seilacher, 1993; Tanabe et al., 2015). The above differences in preservation of the organic jaw material probably result from the location of the redox boundary very close to the sediment-water interface during the sedimentation of chalk during intervals A and B and its significant lowering during chalk deposition of interval C.

The aptychi rarely occur as complete plates. This is the case for some double-valved specimens with apical parts intact (e.g., SI-Fig. 6A,B). In the majority of isolated specimens in hand the apical parts are missing (e.g., Figs. 1c, SI-Fig. 6E,F). The apical parts are the thinnest sectors of aptychi, and break off easily. Some of the damage originated prior to final burial of the specimens as evidenced by incomplete specimens embedded in chalk. However, most of the damage occurred during recovery of specimens from the chalk. We failed to collect and/or recover complete isolated specimens and this is why all specimens used here for geochemical analyses are preserved without the apical portions.

The preservation of large numbers of thin and fragile aptychi and their common articulation point to limited transport and relatively rapid burial at the site where their bearers lived. The aptychi reached the sea-floor either as predator-produced regurgitates (compare Hoffmann et al., 2019) or by becoming separated from the shells following decay of soft tissues. No remains of the entirely organic scaphitid upper jaws, which are much smaller than the lower ones (Landman et al., 2012), have been recorded from Chełm. In view of the fact that the upper jaws of ammonoids have a much lower preservational potential than the lower ones (Tanabe et al., 2015), their absence at Chełm can probably be explained as preservational bias, and not as the result of hydraulic segregation of the jaw elements.

In summary, the taphonomic observations on scaphitid moulds and aptychi from Chełm indicate that these ammonoids lived and were buried in the same habitat, usually a short time after death. Any significant lateral post-mortem transport of these remains from distant habitats

can be ruled out. In other words, the aptychi provide stable isotope signals of the local environment, and are therefore suitable for the present study (compare Sessa et al., 2015).

Interpretation of predation marks

Fossil traces of durophagous predation on invertebrate shells provide data on predator-prey interactions in past communities. Quantitative proportions between various types of such marks in ammonoid assemblages may provide insights into the habitat depth preferences of the ammonoid prey (e.g., Keupp, 2006; Mironenko, 2017, 2020). There are two major types of predation marks in the fossil record: 1) sublethal injuries – repaired (healed) during the life of the prey, and 2) lethal injuries, lacking traces of repair.

No sublethal injuries were identified on the moulds of *Hoploscaphites constrictus* from Chełm. In fact, only a single sublethal (repaired) predation mark has been reported to date from a steinkern of this species from the upper Maastrichtian opokas of the Middle Vistula River section, central Poland (Machalski, 2021). It was inflicted on the aperture of a subadult stage of *H. constrictus crassus*, probably by a squid or nautiloid, as can be determined from its triangular outline (*typus acutus* of Kröger, 2000).

As far as lethal traces on *H. constrictus* are concerned, they are fairly numerous at Chełm as well as in other Maastrichtian successions in Poland (Machalski and Malchyk, 2018). Two types of lethal predation marks occur on moulds of *H. constrictus* from Chełm (SI-Fig. 8).

Ventral injuries. These are represented by subcrescentic or V-shaped notches on the ventrolateral sector of the shell, predominantly near the base of the body chamber (SI-Fig. 8C). This can be assigned to the ichnogenus *Bicrescomanducator* Donovan, Paul, Andrew & Howe, 2010, established on the basis of Early Jurassic ammonoids from Lyme Regis (Andrew et al., 2010). Such traces, discernible in shells of many Mesozoic ammonoids, are interpreted as the result of surprise predatory attacks by fish, marine reptiles, coleoids and other cephalopods on the posterior of ammonoids swimming in the water column (Larson, 2002; Klompmaker et al., 2009; Takeda et al., 2016). These predators could live high in the water column or near the sea floor, so the ventral predation marks are neutral as far as determination of habitat depth preferences of the ammonoid prey is concerned.

Lateral injuries. These are represented by subcircular to irregular holes on the flanks of the body chamber, either on one or on both sides of the shell (SI-Fig. 8B). Pether (1995) assigned such traces to the ichnogenus *Belichnus* Pether, 1995. These distinctive traces were described for the first time on moulds of *Hoploscaphites constrictus crassus* from the upper Maastrichtian of the Middle Vistula River section by Radwański (1996). That author illustrated a suite of specimens with lateral holes on both sides of their body chambers and interpreted these traces as the result of the unsuccesful predation attempts by decapod crustaceans which tried to catch and pinch ammonoid shells from both flanks with their major chelae. Subsequently, the injures described by Radwański (1996) were interpreted as having been inflicted by swimming crabs by Fraaye (1996, p. 272), who envisioned that the lateral holes were produced by such crabs 'attacking from behind, cutting holes in the ammonoid shell at both muscle attachments places, pulling the ammonoid animal out of *his* [sic!] shell and having a nice meal…'. Larson (2002) described lateral holes in shells of US Western Interior scaphitids, favouring their formation by benthic decapod crustaceans, while Keupp (2006) recorded similar sublethal (healed) injuries of this type (his *forma fenestra* type 2) from the shells of Jurassic and Cretaceous ammonoids. The latter author pointed to stomatopods ('mantis shrimps') as the most probable perpetrators of these traces, rejecting their formation by predatory vertebrates or crabs.

Although Machalski and Malchyk (2018) favoured the idea of swimming crabs as makers of the lateral holes, we are convinced now that they were made by stomatopods, in concert with Keupp (2006). The key argument for this interpretation stems from the fact that – contrary to observations made by Radwański (1996) – lateral traces commonly occur only on one side of the scaphitid individuals. Bilaterally preserved specimens of *H. constrictus*, i.e. those with both flanks of the body chamber exposed for observation, are rare at Chełm. However, better data have now been provided by a collection of bilaterally-preserved moulds of *H. constrictus crassus* with identical lateral holes from outcrops at Nasiłów, Bochotnica and Kazimierz Dolny (Middle Vistula River section). Amongst 31 examined specimens bearing the lateral traces in this collection, 18 revealed the presence of these marks on one side of the body chamber only. Moreover, size, shape, number and position of particular holes on the flanks of individuals attacked from both sides commonly differ significantly from each other. This is exemplified by the specimen illustrated in figure 1 in Radwański (1996) which bears a single hole on one flank, and three holes of different size and shape on the opposite flank. Swimming crabs could have been expected to have used both claws as an antagonistic system to grasp and puncture the shells of scaphitids. This would result in the formation of broadly similar and symmetrically located holes on both sides of the preyed shells. This is not the case and we therefore have to exclude grasping predators as producers of the lateral predation marks on the shells of *H. constrictus* from the Polish Maastrichtian. It is better to seek among animals that are able to produce holes on one side of the attacked shells without an 'anvil' on the opposite side. Stomatopods meet this requirement as they produce single 'ballistic' traces on molluskan shells with their smashing or spearing raptorial appendages (Geary et al., 1991; Bałuk and Radwański, 1996; Keupp, 2006). The reader is referred to Caldwell and Dingle (1976) and deVries (2017) for detailed data on the predatory appendages and behavior of Recent stomatopods.

Stomatopod body fossils are extremely rare, being known since Late Jurassic, mostly from exceptional conservation deposits, such as the Solnhofen Plattenkalk, due to their poor fossilisation potential (Keupp, 2006). Admittedly, no stomatopod fossils have been found to date at Chełm; the same holds true, however, for remains of swimming crabs. Recent stomatopods are exclusively benthic predatory crustaceans, and the same habit and habitat may be safely attributed to their fossil representatives (Keupp, 2006). Therefore, the occurrence of stomatopod predation marks on the specimens of *H. constrictus* from Chełm provides evidence that these ammonoids lived near, or at least temporally approached, the sea floor and there fell victim to stomatopod predators.

An alternative explanation for the origin of lateral injuries discussed here is that they result from stomatopod predation on permanent secondary inhabitants of, or just occasional hiders in, the empty ammonoid shells. There has been a growing number of reports on remains of organisms entombed inside ammonoid shells, ranging from decapod crustaceans to fish (e.g., Fraaye and Jäger, 1995; Fraaije, 2003; Fraaije et al., 2020; Mironenko, 2020). As far permanent dwellers of empty ammonoid shells are concerned, we reject this possibility for the Chełm material, based on the lack of any epizoic organisms on stomatopod-infected individuals of *H. constrictus* from this locality. Such epizoans occur occassionally on non-infected specimens, especially from interval C. An extra overgrowth by epizoans would be expected e.g., from the pagurized ammonoid conchs that were dragged for a longer time across the sea floor. As to occasional hiders, we intuitively feel that the simplest way to chase the hiding animals was *via* the open aperture of the empty scaphitid conch. Additionally, occasional regeneration of the lateral traces reported by Larson (2002) and Keupp (2006) points to their origin during the life of the ammonoids. At Chełm, no cases of healing are reported but – as mentioned earlier – sublethal traces on *H. constrictus* are extremely rare in the Maastrichtian of Poland, possibly due to preservational issues (Machalski, 2021).

Frequency of predation marks. This fluctuates between the sampled intervals at Chełm (Fig. 4c and SI Table 1). Of special interest is a marked increase in frequency of lateral (stomatopod-inflicted) traces in sampling interval C, up to 66% of all individuals studied, in comparison to 32% in A, and 22% in B (Fig. 4c). Another aspect is highlighted by the ratio between lateral and ventral traces at successive levels. This may be obtained by dividing the percentage of specimens with lateral marks by the percentage of specimens with ventral bites. This ratio equals 1.16 for A, 1.39 for B, and rises to 8.25 in C (SI Table 1). This means a much higher incidence of benthic predation at this interval, suggesting that *H. constrictus* aff. *crassus* recorded from interval C spent more time near the bottom than individuals of *H. c. lvivensis* from intervals A and B. Statistical testing of data remains inconclusive: there is no clear relationship between types of predation marks and their frequency on scaphitid specimens in the sampled intervals (Kruskal-Wallis test, Hc = 1.143, p = 0.56).

SI Table 1. Data on predation marks on scaphitid moulds from sampled intervals A–C at Chełm:

Interval	A	B	C
Lateral/ventral bite marks	1.16	1.39	8.25
% shells with bite marks	54	58	89

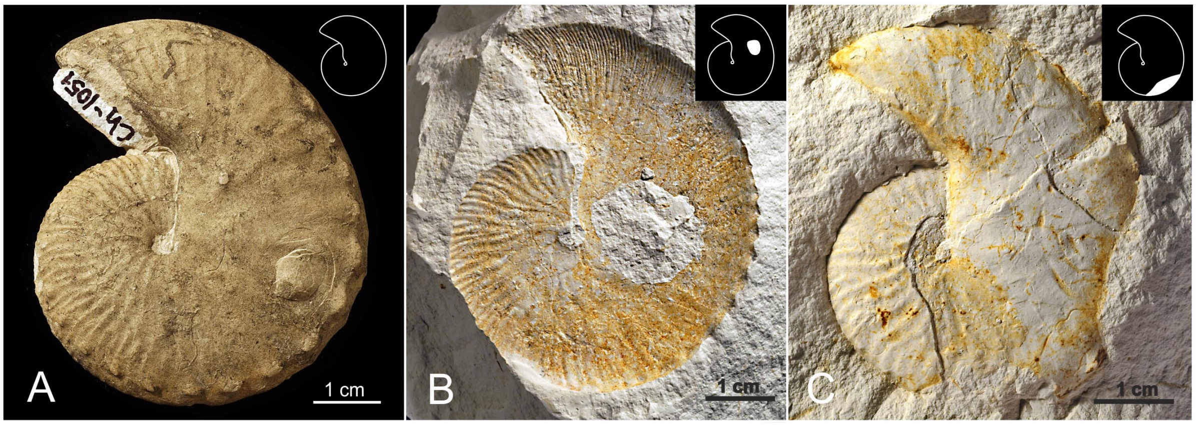

SI Figure 8. (**A**) Intact specimen of *Hoploscaphites constrictus lvivensis*, holotype ZPAL Am. 12/1051 from interval A or B. (**B**) Lateral mark on *H. c.* aff. *crassus*, ZPAL Am. 12/1751, interval C. (**C**) Ventral mark on *H. c. lvivensis*, ZPAL Am. 12/1710, interval B. Silhouettes in top right-hand corner of each photo are used in Figure 3C to symbolize each preservational category of the scaphitid moulds studied.

Evaluation of diagenetic alteration of aptychi

Prior to interpretation of the stable isotope signals from the ammonoid skeletal elements (shells, aptychi), they should be evaluated in terms of possible diagenetic alteration, which would potentially skew their original biological signatures (see e.g., Moryia et al., 2003, Kruta et al., 2014, and Sessa et al., 2015). To assess the stable isotope integrity of the aptychi, we have relied on the following observations.

Firstly, the aptychi studied show well-defined growth increments, in the form of intervening ridges and grooves, on the surface of their concave, anatomically dorsal side (see SI-Fig. 9A; compare Machalski, 2021). The presence of growth increments on the concave, not convex, surface of the aptychi reflects the fact that the successive aptychus layers were laid down on their convex, anatomically ventral surface (Fischer and Fay, 1953, fig. 1; Dzik, 1981; Seilacher, 1993; Machalski, 2021). Distinct growth increments are visible in sections of aptychi, made perpendicular to their surface and approximately along the line of maximum growth of aptychus. The overlapping calcitic lamellae are ~10μm thick and are separated by growth lines which are best visible in plane and cross-polarized light (SI-Fig. 10). The microstructure of aptychi is also well discernible under SEM. These reveal well-defined increments and growth lines (SI-Fig. 9B,C) and pristine lamellar microstructures with distinct lamellar tablets without signs of diagenetic secondary phases (SI-Fig. 9D). All these observations allow us to assign a high Preservation Index 5 to the aptychi studied (excellent preservation, see Kruta et al., 2014).

Secondly, most of the aptychi examined by us with CL microscopy show no luminescence at all (e.g., SI-Fig. 10B) or display very dull, fading luminescence (SI-Fig. 10D,F). As little as 10-20 ppm Mn2+ in solid solution is sufficient to produce visually detectable luminescence, if total Fe contents are below about 150 ppm (Machel et al., 1991; Neuser et al., 1996). Lack of luminescence is consistent with the conclusions about pristine carbonate composition of the specimens studied. The fading luminescence of some specimens can be associated with the presence of organic matter between increments. Organic matter is a sink of many trace elements, including all known activators, sensitizers, and quenchers of CL in carbonates (e.g., Machel, 2000). An exception is specimen ZPAL Am. 24/75 (SI-Fig. 10G,H) recovered from the colluvium in a close proximity of a fissure in the chalk. This aptychus shows strong neomorphism of secondary calcite obliterating microstructure and shares strong homogeneous luminescence with the sediment and a foraminiferal test (SI-Fig. 10 H).

Thirdly, the results of Electron Microprobe (EMPA) analyses suggest trace element content (Mg, S, P, Fe) linked to the biomineralization process of low-magnesium calcite of aptychi, with Mn, Sr, Ba content below detection limit of microprobe (SI Table 2). Detectable amounts of these elements would indicate diagenetic alteration of samples.

Fourthly, the ontogenetic stable isotope profiles of aptychi selected from sampling intervals A–C (Fig. 5, SI-Figs. 15–16) reveal oscillating values of $\delta^{18}O$ which may be interpreted as reflecting seasonal biological cycles; $\delta^{13}C$ values increase during ontogeny (SI-Fig. 15**)**. Similar trends were observed in extant cephalopods (Auclair et al., 2004; Rexfort and Mutterlose, 2009; Guerra et al., 2010) and their presence in fossils have been used as argument for pristine preservation of *in-vivo* isotopie signatures (Fatheree et al., 1998; Lukeneder et al., 2010; Stevens et al., 2015; Ellis and Tobin, 2019; Ferguson et al., 2019). Moreover, isotope signatures both from bulk and serially sampled aptychi plotted against isotope compositions of associated samples of chalk show different values (SI-Figs. 13,14). Diagenesis tends to homogenize seasonal signals, as it resets the original isotope values to those of the surrounding sediment and pore fluids. Therefore, biogenic carbonates characterized by isotope values that vary significantly from the isotope signatures obtained from corresponding sediment samples may be regarded as preserving original bio-signatures.

In summary, the effects of possible diagenetic alteration upon the aptychus and matrix samples has been assessed using petrographic and geochemical data. Relatively low Mn and Fe concentrations and higher Mg levels, in conjunction with petrographic analysis showing low levels of luminescence, confirm that the aptychi experienced little or no diagenetic alteration. Accordingly, they are treated here as reliable sources of biologically meaningful isotope and palaeotemparature data.

SI Table 2. Electron microprobe analyses of selected aptychi and adjacent sediment with foraminiferal tests; N – number of analytical points; foram – foraminifer; b.d.l. – below detection limit:

	Am 24/75 (N=11)	Am 24/74 (N=11)	Am 24/68 (N=10)	Am 24/76 (N=11)	Am 24/75 sediment	Am 24/75 foram	Am 24/76 sediment	Am 24/76 foram
Mg	0.139	0.161	0.1705	0.11	0.172	0.166	0.157	0.011
Si	0.029	b.d.l.	b.d.l.	0.027	0.037	0.137	0.037	0.076
Ca	38.202	36.206	39.173	35.384	38.511	37.441	34.662	34.732
Ba	b.d.l.	b.d.l.	b.d.l.	b.d.l.	b.d.l.	b.d.l.	b.d.l.	b.d.l.
Al	0.058	0.055	b.d.l.	0.016	0.068	0.32	0.522	0.118
P	0.049	0.039	0.0365	0.033	0.045	0.034	b.d.l.	b.d.l.
S	0.093	0.048	0.049	0.056	0.053	0.038	0.085	0.035
Fe	b.d.l.	b.d.l.	b.d.l.	b.d.l.	b.d.l.	1.014	b.d.l.	b.d.l.
Mn	b.d.l.	b.d.l.	b.d.l.	b.d.l.	b.d.l.	b.d.l.	b.d.l.	b.d.l.
Sr	b.d.l.	b.d.l.	b.d.l.	b.d.l.	b.d.l.	b.d.l.	b.d.l.	b.d.l.

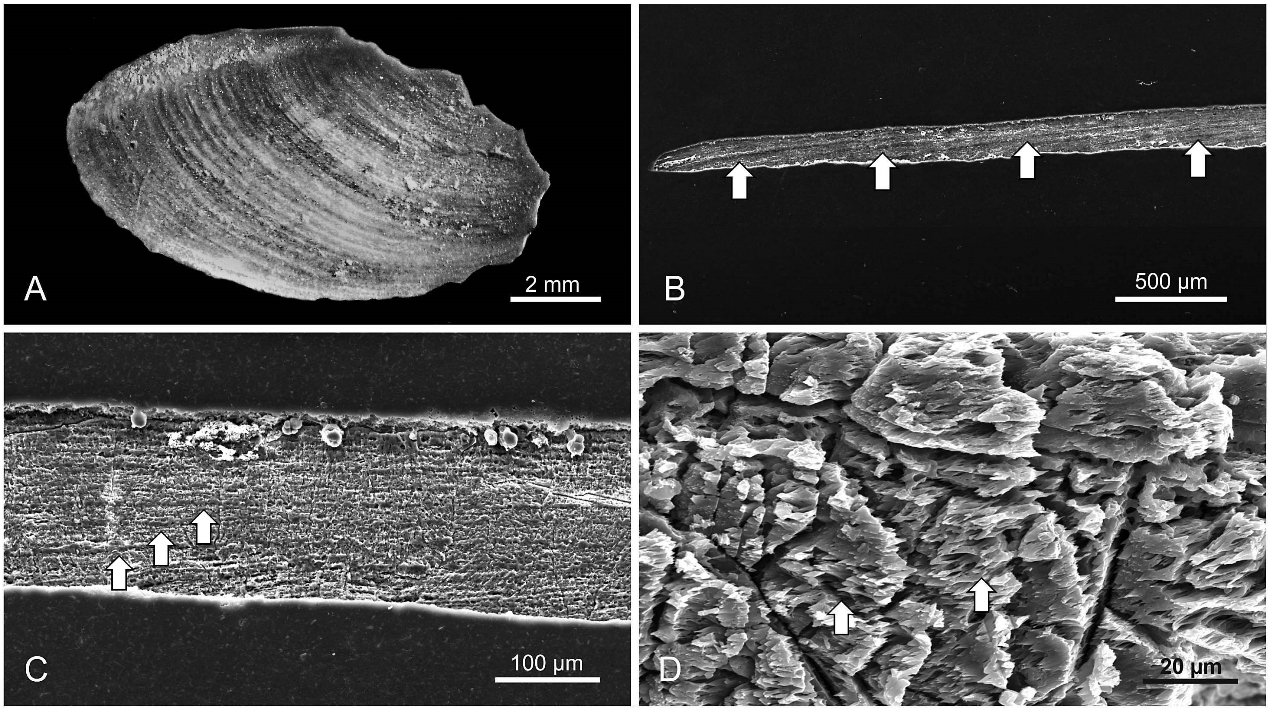

SI Figure 9. Aptychus ZPAL Am. 24/22 from interval B at Chełm. (**A**) Dorsal view of incomplete right valve with growth increments (apical part of specimen broken off). (**B–D**). SEM micrographs of acid-etched polished section of the specimen. The section is perpendicular to the surface of the aptychus and approximately parallel to its maximum growth axis (from right to left; see Machalski, 2021, fig. 2 for explanations of aptychus growth directions and morphology). (B,C). Micrographs showing well-preserved calcitic increments (arrows) and intervening growth lines. (D) Interlocking platelets of low-Mg calcite (arrows).

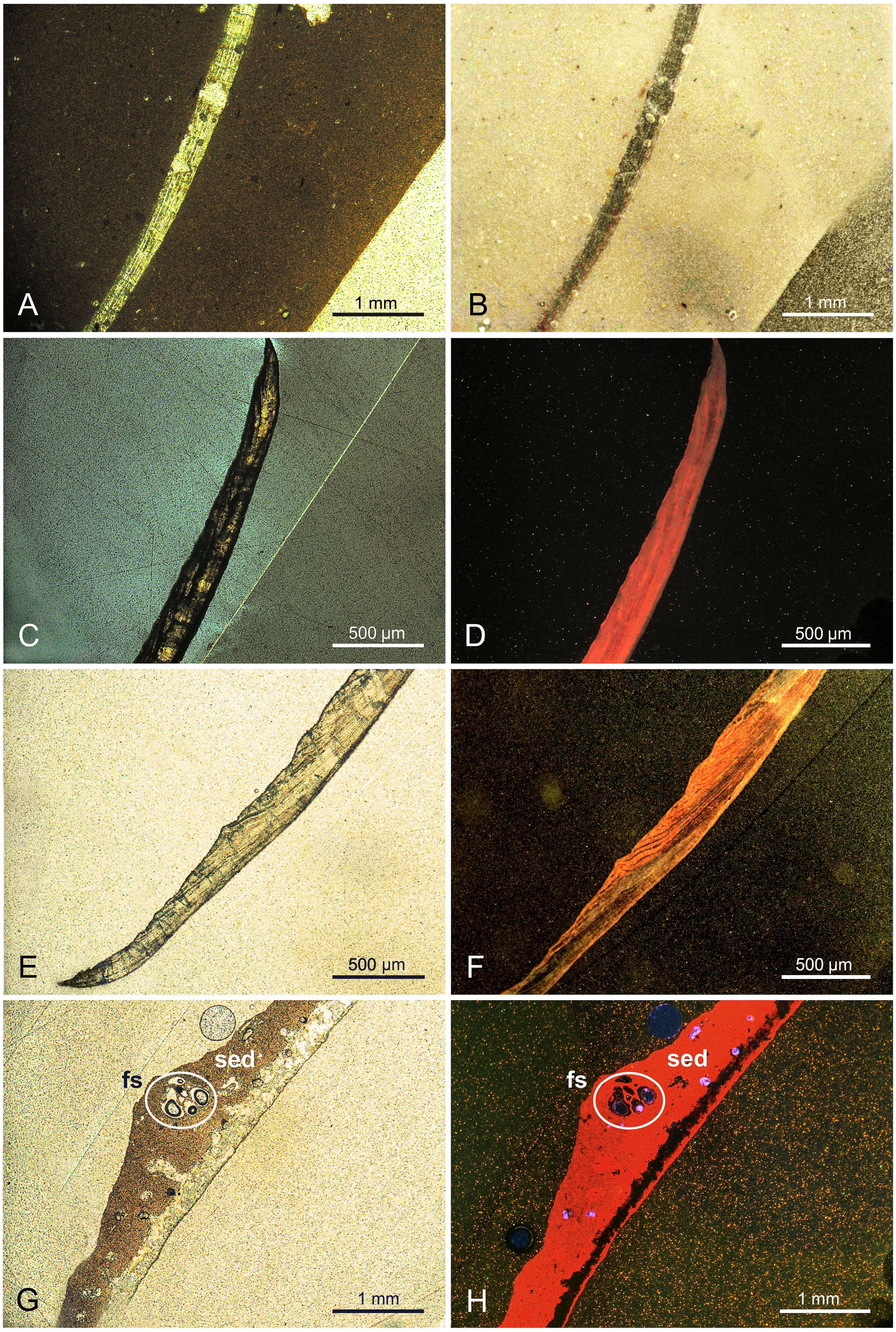

SI Figure 10. Photomicrographs of thin sections containing aptychi under plane-polarized light (**C,E**); cross-polarized light (**A,G**); and cathodoluminescence (**B,D,F,H**). Note weak fading red luminescence at D and F due to presence of organic carbon in aptychus structure, H shows strong red luminescence of aptychus, foram shell (fs) and surrounding sediment (sed) caused by significant Mn ions uptake during diagenesis. Specimen numbers: (A,B) ZPAL Am. 24/76 from interval C; (C,D) ZPAL Am. 24/68 from interval B; (E, F) ZPAL Am. 24/74 from B; (G, H) ZPAL Am. 24/75 from A. Photomicrographs A,C,E,G were scanned using a Nikon Eclipse 80i transmitted light microscope and photomicrographs B,D,F,H were scanned using a HC1-LM hot cathode microscope (see also Materials and methods).

Selection of foraminifera

Foraminiferal tests from the Chełm chalk are of pristine preservation as documented by Dubicka et al. (2018), based on comprehensive analysis including cathodoluminescence, elemental contents and micro/nanno-structure observations.

Two benthic foraminiferal species, *Gyroidinoides globosus* and *Cibicoidoides voltzianus*, and two planktic ones, *Heterohelix striata* and *Globigerinelloides prairiehillensis*, were chosen for comparison of the stable isotope signals they provide to those from co-occurring aptychi of *Hoploscaphites constrictus*. These taxa were documented by Dubicka et al. (2018) to precipitate oxygen and carbon isotopes in near-equilibrium with surrounding water: *G. globosus*, *H. striata* and *G. prairiehillensis* for oxygen and *C. voltzianus* and *G. prairiehillensis* for carbon isotopes. As far as the planktic foraminifera are concerned, the selected taxa represent two different behaviors, that is surface-water dwelling for *Heterohelix* and a shallow-intermediate planktic mode of life for *Globigerinelloides* (Leckie, 1987; Nederbragt et al., 1998). Therefore, they are expected to display isotope values of water surface and slightly deeper in the water column. Foraminiferal species selected in this paper for stable isotope analyses are illustrated in SI-Fig. 11.

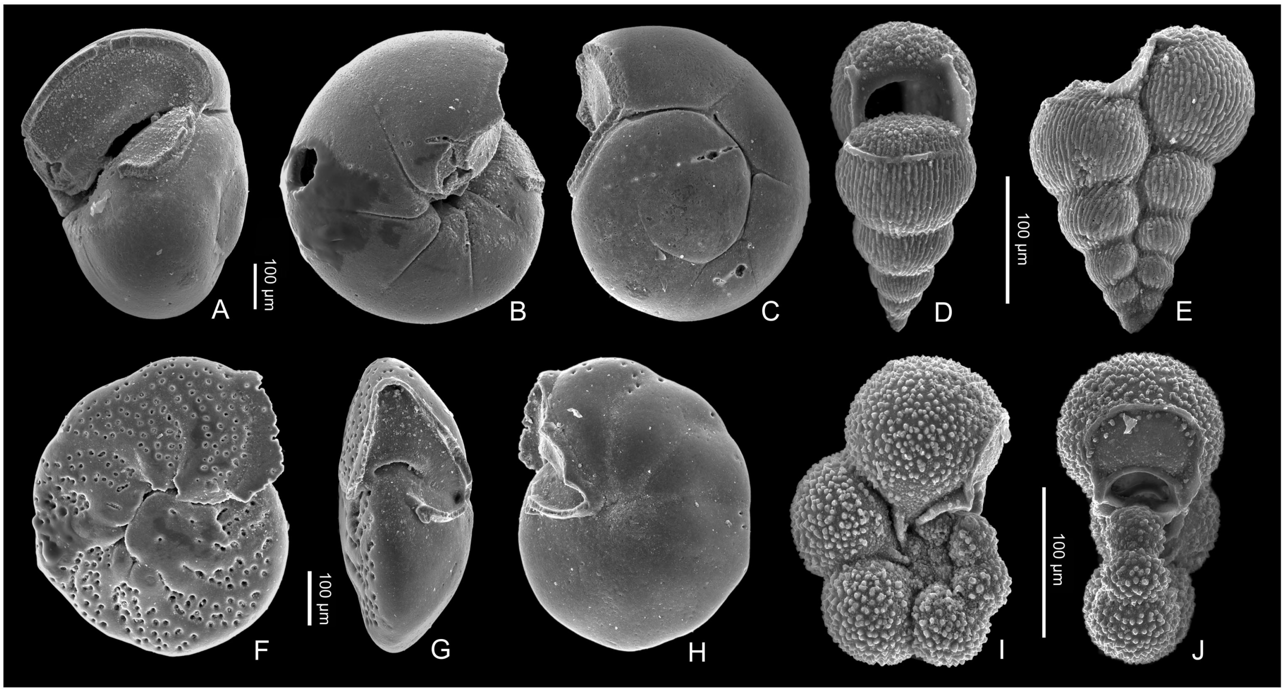

SI Figure 11. SEM images of benthic (**A–C, F–H**) and planktic foraminifera **(D–E, I–J)** species from Chełm used for stable isotope analyses. (A–C) *Gyroidinoides globosus* (Hagenow, 1842). (D–E) *Heterohelix striata* (Ehrenberg, 1840). (F–H) *Cibicidoides voltzianus* (d'Orbigny, 1840). (I–J) *Globigerinelloides prairiehillensis* Pessagno, 1967.

Stable Isotope data

Additional diagrams

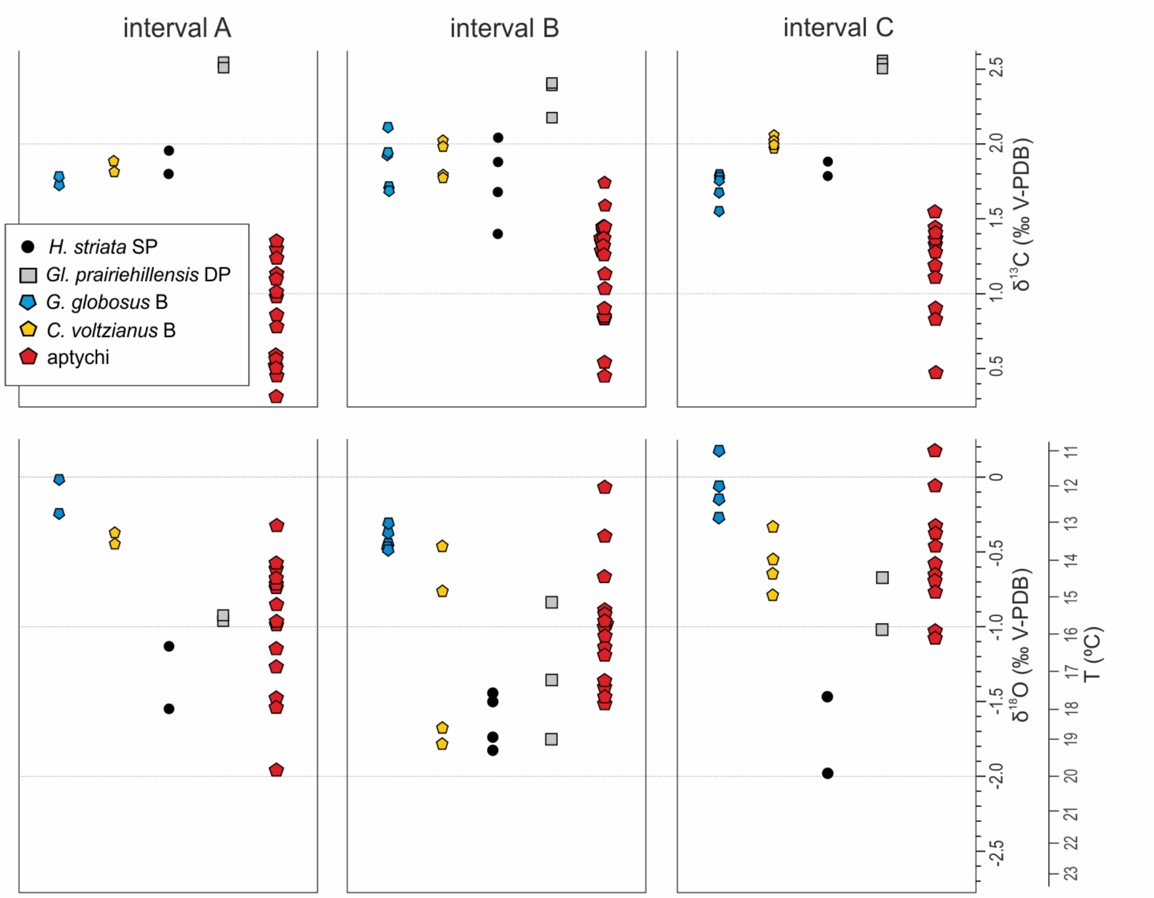

SI Figure 12. Variations in δ13C and δ18O values of aptychi and selected benthic and planktic foraminiferal species from the intervals A–C at Chełm. Abbreviations: *H.*, *Heterohelix*; *Gl.*, *Globigerinelloides*; *G.*, *Gyroidinoides*; *C.*, *Cibicidoides*.

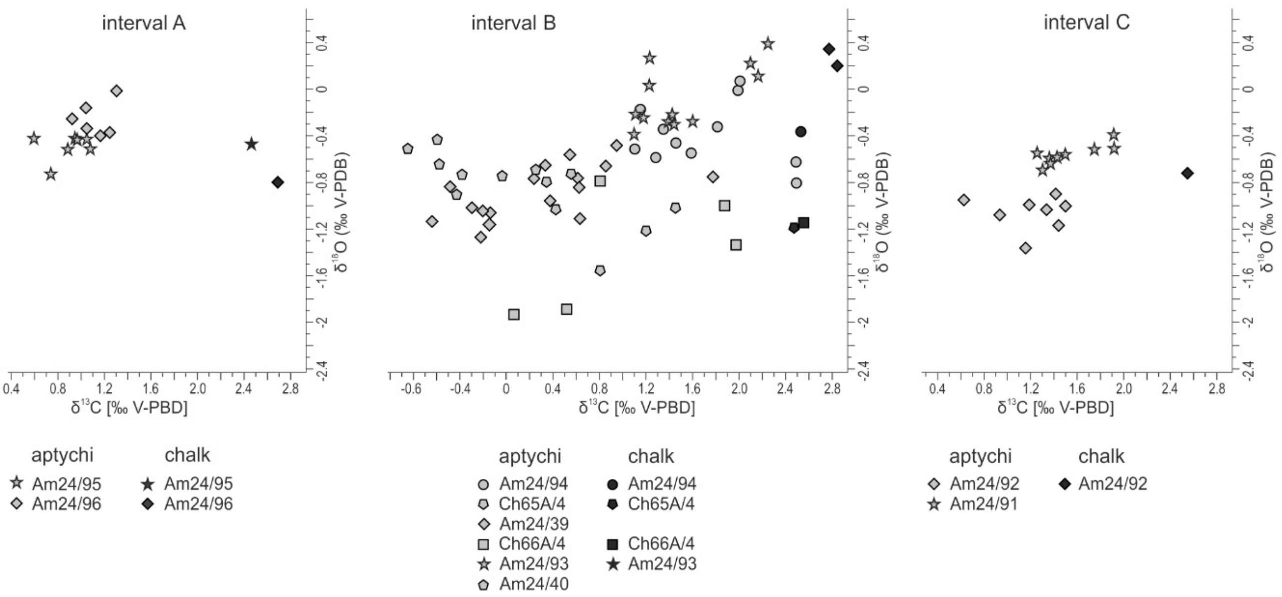

SI Figure 13. Carbon isotope compositions plotted against their corresponding oxygen isotope compositions based on serially sampled aptychi and adjacent chalk from intervals A–C at Chełm.

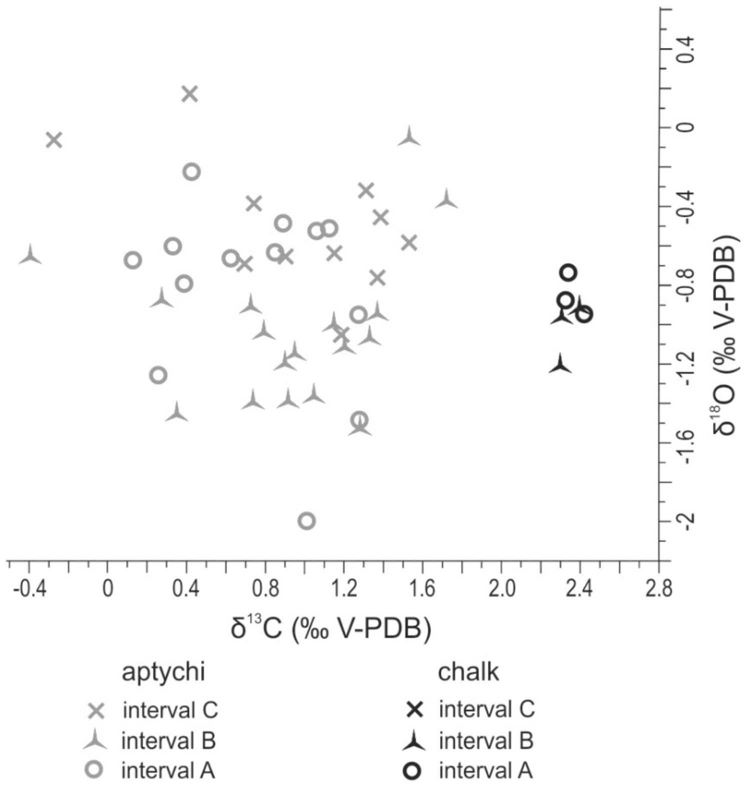

SI Figure 14. Carbon isotope compositions plotted against their corresponding oxygen isotope compositions of bulk aptychi and adjacent chalk from intervals A–C at Chełm.

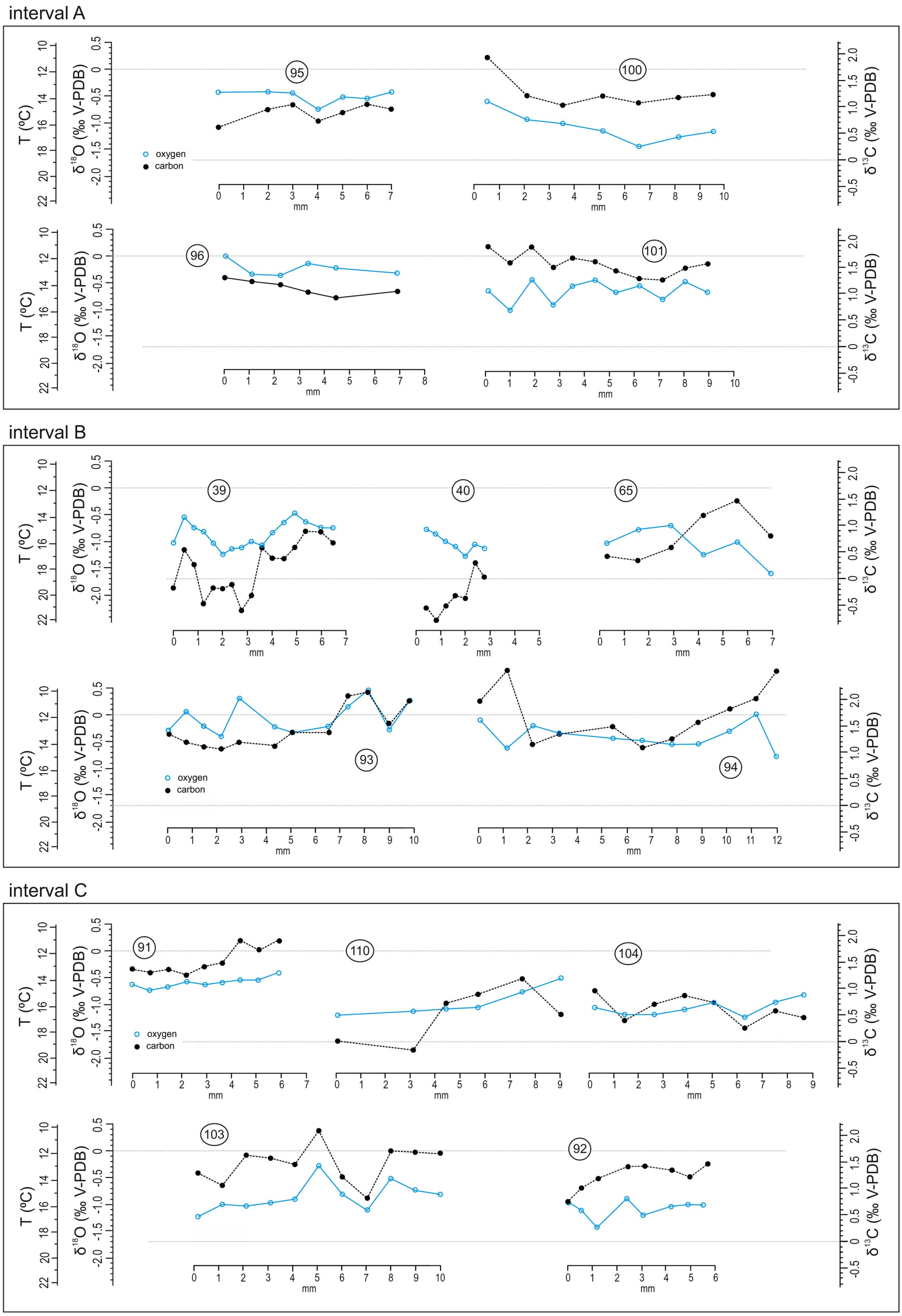

SI Figure 15. Stable isotope curves based on serial sampling of selected aptychi from intervals A–C at Chełm (each series of measurements follows the growth direction of the aptychus, which is from the left to right; see also Figure 5).

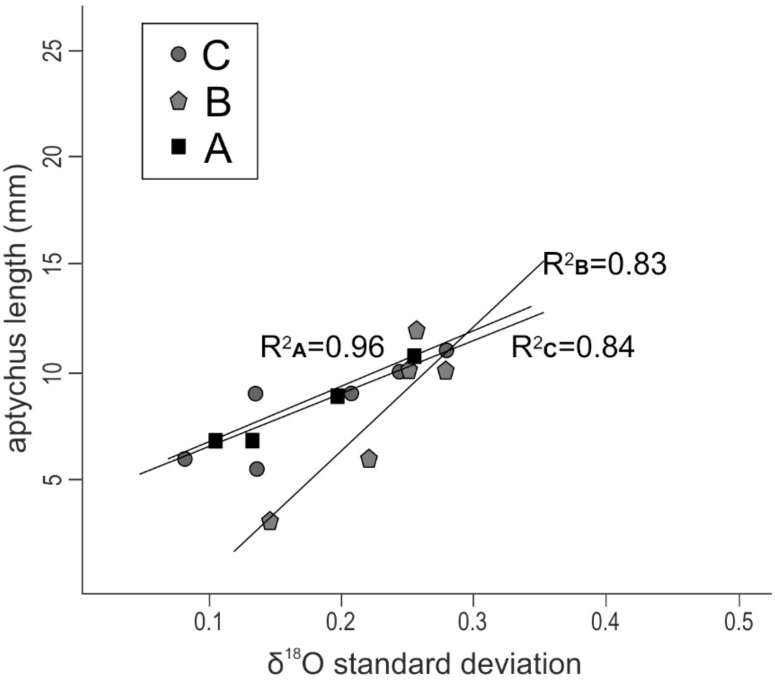

SI Figure 16. Aptychi length (mm) plotted against their corresponding intra-specimen standard variation in oxygen isotope compositions from intervals A–C at Chełm. The near-linear relationship (reflected in regression coefficients >0.8) shows that aptychi age/size (reflecting preservation of more complete annual cycles) is responsible for the variability of isotope signatures in examined specimens.

Tables with rough isotope and palaeotemperature data

SI Table 3. Isotope mean values for targeted aptychi and foraminifera from sampling intervals A–C at Chełm; data used in Figs. 2 and 3:

Sampling interval	A	B	C
	δ^{13}C (‰V-PDB)		
G. prairiehillensis	2.58	2.35±0.12	2.58±0.07
Aptychi of *H. c.*	0.72±0.38	1±0.45	0.87±0.55
C. voltzianus	1.87±0.02	1.94±0.09	2.02±0.0.05
	δ^{18}O(‰V-PDB)		
H. striata	-1,34±0.22	-1.62±0.18	-1.71±0.0.25
G. prairiehillensis	-0.875±0.02	-1.33±0.46	-0.77±0.0.27
Aptychi of *H. c.*	-0.92±0.45	-1.05±0.34	-0.48±0.35
G. globosus	-0.1±0.12	-0.41±0.09	-0.9±0.28

SI Table 4. Mean values of palaeotemperatures (in °C) calculated from targeted foraminifera and aptychi from sampling intervals A–C at Chełm; data used in Figs. 2 and 3:

Sampling interval	A	B	C
H.striata	17.2±1	18.4±0.8	18,82±1.5
G. prairiehillensis	15.3±0.06	17.2±1.9	14.6±1.4
Aptychi of *H. Constrictus*	15.0±0.5	16.0±0.3	13.8±0.9
G. globosus	12.2±0.5	13.3±0.3	12.2±1.2

SI Table 5. Isotopes values for serially sampled aptychi from interval C; data used in SI-Figs. 12 and 14; SD – standard deviation:

Specimen	$\delta^{13}C$ (‰ V-PDB)	SD [‰]	$\delta^{18}O$ (‰ V-PDB)	SD [‰]
Am24/91	1.36	0.03	-0.61	0.03
Am24/91	1.31	0.02	-0.71	0.04
Am24/91	1.37	0.04	-0.64	0.05
Am24/91	1.26	0.02	-0.55	0.05
Am24/91	1.42	0.03	-0.60	0.03
Am24/91	1.49	0.02	-0.57	0.02
Am24/91	1.90	0.02	-0.52	0.03
Am24/91	1.74	0.02	-0.53	0.04
Am24/91	1.89	0.03	-0.40	0.10
Am24/92	0.64	0.02	-0.95	0.06
Am24/92	0.95	0.01	-1.09	0.05
Am24/92	1.16	0.03	-1.36	0.08
Am24/92	1.42	0.03	-0.90	0.02
Am24/92	1.43	0.02	-1.17	0.02
Am24/92	1.34	0.03	-1.04	0.05
Am24/92	1.19	0.04	-1.00	0.02
Am24/92	1.49	0.03	-1.00	0.04

SI Table 6. Isotope values for serially sampled aptychi from interval B; data used in SI-Figs. 12 and 14:

Specimen	$\delta^{13}C$ (‰ V-PDB)	SD [‰]	$\delta^{18}O$ (‰ V-PDB)	SD [‰]
Am24/93	1.40	0.03	-0.27	0.06
Am24/93	1.23	0.01	0.04	0.05
Am24/93	1.15	0.02	-0.21	0.02
Am24/93	1.10	0.01	-0.38	0.05
Am24/93	1.24	0.04	0.27	0.08
Am24/93	1.17	0.02	-0.22	0.06
Am24/93	1.44	0.02	-0.31	0.04
Am24/93	1.43	0.03	-0.21	0.11
Am24/93	2.17	0.01	0.12	0.03
Am24/93	2.26	0.01	0.39	0.12
Am24/93	1.61	0.03	-0.27	0.03
Am24/93	2.10	0.01	0.22	0.05
Am24/94	2.00	0.02	-0.01	0.07
Am24/94	2.50	0.02	-0.62	0.02
Am24/94	1.15	0.01	-0.17	0.07
Am24/94	1.35	0.02	-0.34	0.03
Am24/94	1.46	0.03	-0.46	0.04
Am24/94	1.11	0.03	-0.52	0.02
Am24/94	1.29	0.03	-0.58	0.05
Am24/94	1.59	0.02	-0.55	0.03
Am24/94	1.82	0.02	-0.32	0.03
Am24/94	2.02	0.01	0.08	0.02
Am24/94	2.50	0.02	-0.80	0.03
Am24/40	-0.04	0.03	-0.75	0.03
Am24/40	0.26	0.01	-0.70	0.02
Am24/40	-0.43	0.02	-0.90	0.05
Am24/40	-0.38	0.03	-0.73	0.04
Am24/40	-0.58	0.01	-0.64	0.03
Am24/40	-0.85	0.03	-0.51	0.02
Am24/40	-0.60	0.02	-0.42	0.02
Am24/39	-0.20	0.01	-1.05	0.04
Am24/39	-0.49	0.02	-0.84	0.10
Am24/39	-0.14	0.02	-1.06	0.04
Am24/39	-0.23	0.02	-1.26	0.03
Am24/39	-0.65	0.01	-1.13	0.05
Am24/39	-0.31	0.01	-1.01	0.03
Am24/39	0.63	0.10	-1.10	0.06
Am24/39	0.95	0.01	-0.48	0.03
Am24/39	0.86	0.01	-0.65	0.04
Am24/39	0.54	0.01	-0.56	0.04
Am24/39	0.23	0.01	-0.75	0.05
Am24/39	-0.15	0.02	-1.15	0.04
Am24/39	0.38	0.01	-0.95	0.04
Am24/39	0.33	0.03	-0.65	0.04

Am24/39	0.63	0.01	-0.84	0.02
Am24/39	0.61	0.00	-0.76	0.06
Am24/39	1.78	0.01	-0.74	0.04
Am24/66	0.06	0.06	-1.93	0.08
Am24/66	1.97	0.06	-1.33	0.11
Am24/66	1.88	0.06	-0.99	0.12
Am24/66	0.51	0.04	-1.89	0.08
Am24/66	0.80	0.04	-0.78	0.09
Am24/65	0.42	0.06	-1.03	0.08
Am24/65	0.34	0.05	-0.78	0.10
Am24/65	0.56	0.05	-0.71	0.11
Am24/65	1.20	0.05	-1.21	0.10
Am24/65	1.46	0.06	-1.00	0.07
Am24/65	0.81	0.04	-1.55	0.10

SI Table 7. Isotope values for serially sampled aptychi from interval A; data used in SI-Figs. 12 and 14:

Specimen	$\delta^{13}C$ (‰ V-PDB)	SD [‰]	$\delta^{18}O$ (‰ V-PDB)	SD [‰]
Am24/95	0.61	0.03	-0.42	0.02
Am24/95	0.94	0.05	-0.42	0.09
Am24/95	1.02	0.03	-0.43	0.06
Am24/95	0.74	0.02	-0.73	0.02
Am24/95	0.88	0.05	-0.51	0.02
Am24/95	1.04	0.03	-0.52	0.04
Am24/95	0.96	0.03	-0.42	0.03
Am24/96	1.25	0.04	-0.02	0.12
Am24/96	1.19	0.02	-0.37	0.04
Am24/96	1.13	0.03	-0.40	0.04
Am24/96	1.02	0.01	-0.17	0.01
Am24/96	0.91	0.03	-0.25	0.05
Am24/96	1.02	0.02	-0.35	0.04

SI Table 8. Isotope values for sediment samples adjacent to serially sampled aptychi from intervals A–C at Chełm; data used in SI-Fig. 12:

Sample adjacent to aptychus no:	$\delta^{13}C$ (‰ V-PDB)	SD [‰]	$\delta^{18}O$ (‰ V-PDB)	SD [‰]
Am24/96	2.49	0.02	-0.8	0.06
Am24/95	2.28	0.03	-0.48	0.03
Am24/94	2.54	0.02	-0.36	0.02
Am24/39	2.79	0.02	0.35	0.03
Am24/39	2.86	0.01	0.20	0.02
Am24/66	2.54	0.05	-1.13	0.11
Am24/65	2.49	0.08	-1.17	0.13
Am24/92	2.52	0.02	-0.71	0.05

SI Table 9. Isotope values for four species of foraminifera and bulk sediment samples from Chełm; data used in Fig. 2; x – no record:

**Sam-ple**	$\delta^{18}O$ (‰V-PDB)	$\delta^{13}C$ (‰V-PDB)	$\delta^{18}O$ (‰V-PDB)	$\delta^{13}C$ (‰V-PDB)	$\delta^{18}O$ (‰V-PDB)	$\delta^{13}C$ (‰V-PDB)	$\delta^{18}O$ (‰V-PDB)	$\delta^{13}C$ (‰V-PDB)	$\delta^{18}O$ (‰V-PDB)	$\delta^{13}C$ (‰V-PDB)
	C. voltzianus		*G. globosus*		*G. prairiehillensis*		*H. striata*		bulk sediment	
**10.V**	-1.44	1.73	-0.32	1.67	-1.00	2.52	-1.69	1.53	-0.99	2.37
**9.V**	x	x	x	x	x	x	x	x	-0.90	2.31
**8.V**	-0.96	1.80	-0.26	1.63	-1.10	2.56	x	x	-0.74	2.33
**7.V**	-0.77	1.87	-0.23	1.62	-1.20	2.80	-3.67	1.63	-1.07	2.27
**6.V**	x	x	x	x	x	x	x	x	-0.96	2.37
**5.V**	-0.96	1.82	-0.54	1.57	-1.02	2.32	-2.70	1.47	-0.81	2.31
**4.V**	x	x	x	x	x	x	x	x	-1.03	2.29
**3.V**	-0.91	1.81	-0.31	1.55	-1.64	2.56	-1.72	1.56	-0.90	2.32
**2.V**	x	x	x	x	x	x	x	x	-0.96	2.41
**1.V**	-0.82	1.68	-0.37	1.62	-1.23	2.60	x	x	-0.80	2.37
**8.IV**	x	x	x	x	x	x	x	x	-1.02	2.36
**7.IV**	-0.79	1.95	-0.42	1.73	-1.76	2.14	-1.50	2.07	-1.15	2.26
**6.IV**	x	x	x	x	x	x	x	x	-0.94	2.36
**5.IV**	x	x	x	x	x	x	x	x	-1.13	2.32
**4.IV**	-0.99	1.78	-0.35	1.7	-1.35	2.4	-2.52	1.40	-1.17	2.32
**3.IV**	x	x	x	x	x	x	x	x	-0.99	2.28
**2.IV**	x	x	x	x	x	x	x	x	-0.91	2.40
**1.IV**	-0.90	2.04	-0.30	1.80	-1.26	2.37	x	x	-0.96	2.36
**6.III**	-0.78	2.08	-0.25	1.76	-1.04	2.51	-1.97	1.80	x	x
**5.III**	x	x	x	x	x	x	x	x	x	x
**4.III**	x	x	x	x	x	x	x	x	-1.05	2.31
**3.III**	-0.93	2.13	-0.37	1.72	-1.56	2.4	-2.19	1.69	-1.19	2.32
**2.III**	x	x	x	x	x	x	x	x	x	x
**1.III**	-1.10	1.98	-0.51	1.78	-1.26	2.19	-2.31	1.23	-1.25	2.35
**6.II**	x	x	x	x	x	x	x	x	-1.02	2.38
**5.II**	x	x	x	x	x	x	x	x	-0.97	2.23
**4.II**	x	x	x	x	x	x	x	x	-1.25	1.93
**3.II**	x	x	x	x	x	x	x	x	x	x
**2.II**	x	x	x	x	x	x	x	x	-1.26	2.01
**1.II**	x	x	x	x	x	x	x	x	-1.10	2.02

SI Table 10. Isotope values for planktic and benthic foraminifera from Chełm; data used in Fig. 2 and SI-Fig. 15; x – no record:

Sample and sampling interval	$\delta^{13}C$ (‰V-PDB)	$\delta^{18}O$ (‰V-PDB)	$\delta^{13}C$ (‰V-PDB)	$\delta^{18}O$ (‰V-PDB)	$\delta^{13}C$ (‰V-PDB)	$\delta^{18}O$ (‰V-PDB)	$\delta^{13}C$ (‰V-PDB)	$\delta^{18}O$ (‰V-PDB)
	Cibicidoides voltzianus		*Gyroidinoides globosus*		*Globigerinelloides prairiehillensis*		*Heterohelix striata*	
6.III **C**	2.08	-0.78	1.76	-0.25	2.51	-1.04	1.80	-1.97
Ch III x-3 **C**	2.01	-0.52	1.51	-0.04	2.59	-0.68	x	x
Ch III x-11 **C**	2.03	-0.31	1.68	0.19	2.63	-0.74	1.89	-1.46
Ch III 64/x-4 **C**	2.01	-0.63	1.65	-0.14	2.61	-0.60	x	x
7.IV **B**	1.95	-0.79	1.73	-0.42	2.14	-1.76	2.07	-1.50
Ch IV 31-47 **B**	1.93	-0.47	1.95	-0.46	2.44	-0.84	1.68	-1.80
Ch IV 48-55 **B**	1.81	-1.80	2.13	-0.44	2.46	-1.4	1.85	-1.46
Ch IV-1 **B**	2.00	-1.70	1.94	-0.32	x	x	1.12	-1.72
Ch V-118 **A**	1.90	-0.45	1.77	-0.24	2.58	-0.86	1.74	-1.56
Ch V-104 **A**	1.85	-0.37	1.65	0.03	2.58	-0.89	x	x
Ch V-12 **A**	x	X	x	x	x	x	1.93	-1.12

SI Table 11. Isotope values for bulk aptychi from interval C; data used in Figs. 2–4 and SI-Figs. 13 and 15; * values averaged from serially sampled specimens:

Sample	$\delta^{13}C$ (‰ V-PDB)	$\delta^{18}O$ (‰ V-PDB)
Am24/91*	1.52	-0.57
Am24/92*	1.20	-1.06
Am24/63	-0.26	-0.05
Am24/62	0.75	-0.37
Am24/64	0.70	-0.68
Am24/57	1.34	-1.03
Am24/59	1.31	-0.32
Am24/61	0.90	-0.63
Am24/60	1.15	-0.63
Am24/58	1.38	-0.44
Am24/56	1.36	-0.75

SI Table 12. Isotope values for bulk aptychi from interval B; data used in Figs. 2–4 and SI-Figs. 13 and 15; * values averaged from serially sampled specimens:

Sample	$\delta^{13}C$ (‰ V-PDB)	$\delta^{18}O$ (‰ V-PDB)
Am24/93*	1.53	-0.07
Am24/94*	1.71	-0.39
Am24/40*	-0.38	-0.66
Am24/39*	0.28	-0.88
Am24/66*	1.04	-1.38
Am24/65*	0.80	-1.05
Am24/16	0.73	-0.92
Am24/34	0.36	-1.47
Am24/7	1.15	-1.02
Am24/2	0.92	-1.19
Am24/24	1.28	-1.53
Am24/3	1.19	-1.10
Am 24/18	1.21	-1.11
Am24/26	0.74	-1.40
Am24/10	1.36	-0.97
Am24/41	1.32	-1.06

SI Table 13. Isotope values for bulk aptychi from interval A; data used in Figs. 2–4 and SI-Figs. 13 and 15; * values averaged from serially sampled specimens:

Sample	$\delta^{13}C$ (‰ V-PDB)	$\delta^{18}O$ (‰ V-PDB)
Am24/95*	0.89	-0.49
Am24/96*	1.06	-0.53
Am24/51	1.28	-0.95
Am24/48	1.02	-2.00
Am24/54	0.33	-0.60
Am24/55	1.13	-0.50
Am24/49	0.26	-1.25
Am24/16	0.73	-0.92
Am24/34	0.36	-1.47
Am24/24	1.28	-1.53
Am24/18	1.21	-1.11
Am24/47	0.44	-0.23
Am24/53	0.40	-0.80
Am24/44	0.86	-0.65
Am24/46	0.13	-0.67
Am24/52	0.63	-0.67

Additional research

Search for Pyrite Framboids. Framboidal pyrite can be used as a proxy for palaeoenvironmental information on water-column and sea-floor oxygenation (Wilkin et al., 1996, 1997; Wignall and Newton, 1998; Bond et al., 2004; Bond and Wignall, 2005, 2010). Pyrite framboids form at the redox boundary within the sediment (iron reduction zone) and stop growing once they reach the sulfate reduction-zone (Bond and Wignall, 2010). Their size and variability are useful tools in defining the redox state of the sea floor. Bond and Wignall (2010) used different combinations of framboidal pyrite sizes and variability to discriminate five redox states, from completely euxinic to weakly dysoxic. During euxinic conditions the framboids start nucleating in the water column and sink out of the redox zone and cease to grow when they reach ~6 μm (Tagliavento et al., 2020). In contrary, in dysoxic environments the framboids form inside the very superficial sediments and are able to grow larger and more variable in size. Short temporal redox variations are often not recorded in the trace fossil assemblages, ichnofabric or benthic faunal response (Lauridsen and Surlyk, 2008; Lauridsen et al., 2011; Tagliavento et al., 2020).

Three samples of chalk from Chełm (one for every sampling interval) were investigated for the presence of pyrite framboids, with negative results. Most of the residue analyzed represents clay minerals, silica, sponges spicules and tiny phosphatized crustacean remains. Therefore, there is no indication of oxygen defficiency, neither in the water column nor at the sea floor during the deposition of the Chełm chalk, which would potentially have influenced the habitat of the ammonoids studied.

REE Analyses. Chalk samples from Chełm studied have the Rare Earth Elements (REE) pattern depleted in light lanthanides relative to medium and heavy REEs, which is reflected in the slightly elevated Yb_N/Nd_N ratios (~1.2). These chalk samples have superchondritic Y/Ho values (40.5 ± 0.5) and negative cerium anomalies (Ce_N/Ce^*_N <0.8) (SI-Fig. 17).

The sample from the upper upper Maastrichtian opoka at Wola Piasecka has more 'shale' REE pattern, which is reflected in the Yb_N/Nd_N ratio = 1. This sample has superchondritic Y/Ho value (32.77) and weak negative cerium anomaly (Ce_N/Ce^*_N =0.84) (SI-Fig. 18).

Recent sea water REE normalized to PAAS exhibit HREE enriched pattern with distinct light rare earth elements (LREE) depletion, negative Ce anomaly, positive La anomaly and high Y/Ho ratios (between 30 and 74; e.g. De Baar et al., 1991; Bau and Dulski, 1996; Byrne and Sholkovitz, 1996; Nozaki, 2001). Pronounced Ce anomalies Ce/Ce*<0.8 in the samples studied are indicative of sediment formation in well-oxygenated waters (Kamber et al., 2014). Moreover, a trend of decreasing Ce/Ce* vs $(La/Ce)_N$ observed in samples from intervals A-C at Chełm and a sample from Wola Piasecka (SI-Fig. 19) suggest successive shallowing of the sea (Zhang et al., 2017).

Sediments deposited in anoxic conditions are generally enriched in redox-sensitive elements such as Mo, U, V and Co (Tribovillard et al., 2006) and have U/Th ratio above 1.25 (e.g., Jones and Manning, 1994; Rakociński et al., 2016). Chalk samples from Chełm contain Mo, U, V and Co concentrations characteristic of carbonate sediments deposited in oxic conditions which is further supported by the low measured U/Th ratios (0.46-0.61). The sample from Wola Piasecka has similar trace element ratios to Chełm samples with only elevated U/Th ratio (0.94). Summarizing, the results show that the sediments from Chełm and Wola Piasecka were deposited largely in oxygenated conditions.

Elements such as Cu, Zn, Cd, Ni and Ba may serve as micronutrients in oxic marine environments, and elevated concentrations of these elements could reflect high palaeo-productivity of the water column (de Tribovillard et al., 2006). All of the above-mentioned elements show a trend of increasing concentrations in the Chełm section which is correlated with

the sea shallowing. The sample from Wola Piasecka has redox-sensitive metals content similar to values from the Chełm samples. Cd/Mo ratios could be differentiated between hydrographically restricted basins (<0.1) and upwelling settings (>0.1; Sweere et al., 2016). Cd/Mo ratios both from both Chełm chalk and Wola Piasecka opoka have high values >1 observed in plankton skeletons (not surprising in chalk which consists mostly of coccoliths). These values, paired with low Co(ppm)xMn (%) <0.4, suggest a high level of primary productivity in the photic zone in a non-restricted/open marine environment (Sweere et al., 2016).

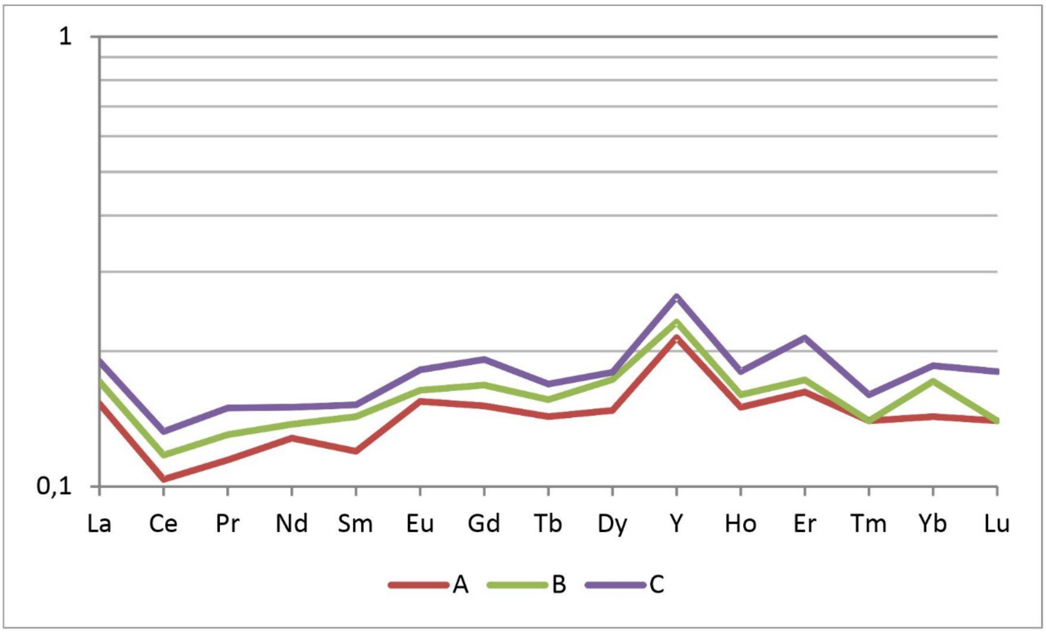

SI Figure 17. PAAS-normalized REE patterns of the lower upper Maastrichtian chalk succession at Chełm, A–C – sampling intervals.

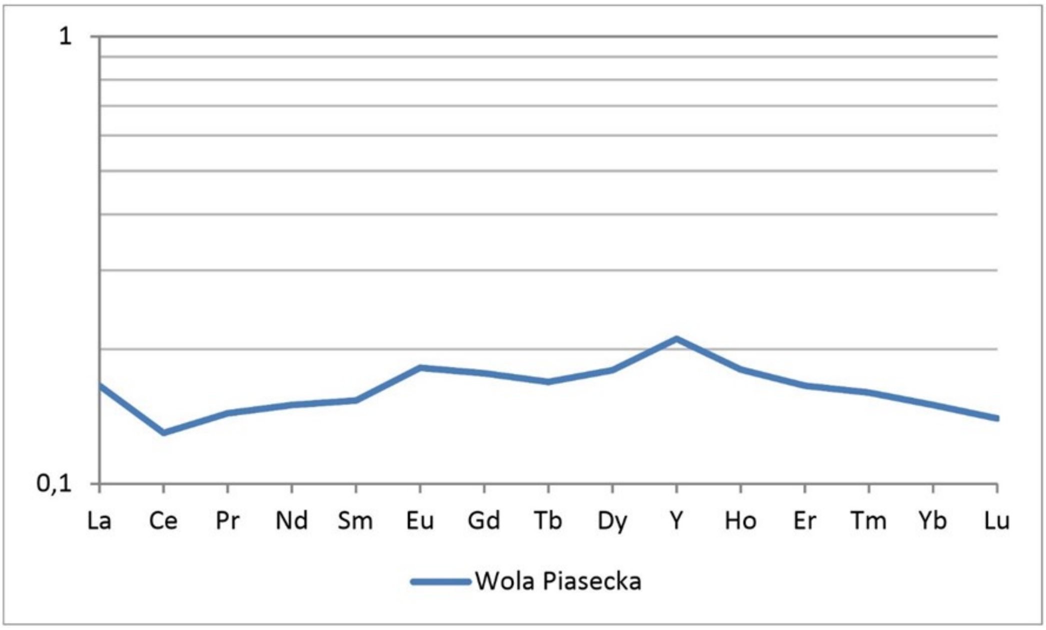

SI Figure 18. PAAS-normalized REE patterns of upper upper Maastrichtian opoka from Wola Piasecka.

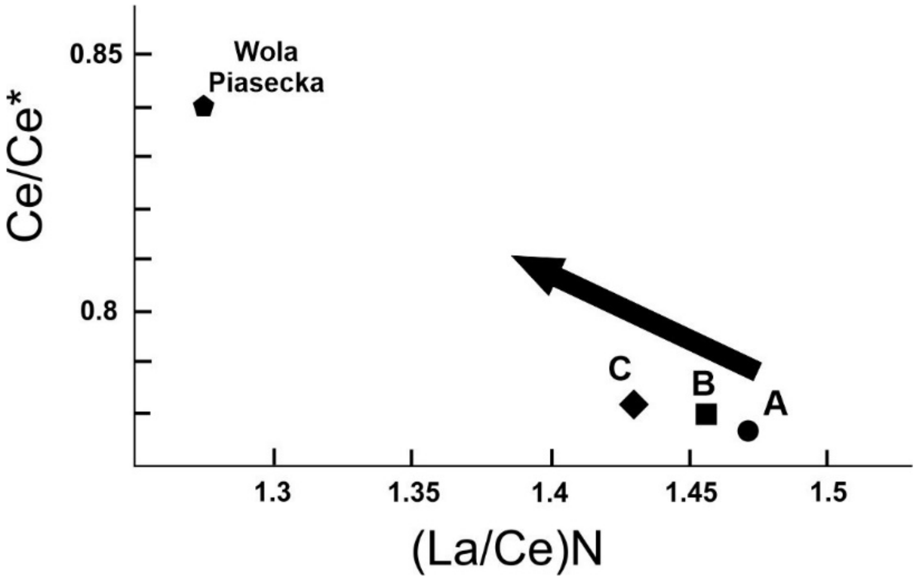

SI Figure 19. Plot of the Ce/Ce* value vs. La/CeN ratios in bulk sediment samples from Wola Piasecka and intervals A–C from Chełm. Note progressive trend from chalk sample A at Chełm to the opoka sample from Wola Piasecka (trend is indicated by an arrow) suggesting progressive shallowing of the marine basin during Maastrichtian.

References

1. Andrew, C., Howe, P., Paul, C.R.C. & Donovan, S.K. Fatally bitten ammonites from the lower Lias Group (Lower Jurassic) of Lyme Regis, Dorset. *Proc. of the Yorksh. Geol. Soc.* **58**, 81–94; https://doi.org/10.1144/pygs.58.1.276 (2010).
2. Auclair, A.-C., Lecuyer, C., Bucher, H. & Sheppard, S.M.F. Carbon and oxygen isotope composition of *Nautilus macromphalus*: a record of thermocline waters off New Caledonia. *Chem. Geol.* **207**, 91–100; https://doi.org/10.1016/j.chemgeo.2004.02.006 (2004).
3. Bałuk, W. & Radwański, A. Stomatopod predation upon gastropods from Korytnica basin, and from other classical Miocene localities in Europe. *Acta Geol. Pol.* **46**, 279–304 (1996).
4. Bau, M. & Dulski, P. Distribution of yttrium and rare-earth elements in the Penge and Kuruman iron-formations, Transvaal Supergroup, South Africa. *Prec. Res.* **79**, 37–55; https://doi.org/10.1016/0301-9268(95)00087-9 (1996).
5. Birkelund, T. Maastrichtian ammonites from Hemmoor, Niederelbe (NW−Germany). *Geol. Jahrb. **A*** **61**, 13–33 (1982).
6. Bitner, M.A. Brachiopods from the Upper Cretaceous white chalk of Chełm, south-eastern Poland. In 9th Paleontological Conference (eds Pisera, A. *et al.*) 9–11 (2008).
7. Bojanowski, M.J., Dubicka, Z., Minoletti, F., Olszewska-Nejbert, D. & Surowski, M. Stable C and O isotopic study of the Campanian chalk from the Mielnik section (eastern Poland): Signals from bulk rock, belemnites, benthic foraminifera, nannofossils and microcrystalline cements. *Palaeogeography, Palaeoclimatology, Palaeoecology* **465**, 193–211; https://doi.org/10.1016/j.palaeo.2016.10.032 (2017).
8. Bond, D.P.G. & Wignall, P.B. Evidence for Late Devonian (Kellwasser) anoxic events in the Great Basin, western United States. In Understanding Late Devonian and Permian-Triassic Biotic and Climatic Events: Towards an Integrated Approach (eds Over, D. *et al.*). *Devel. in Pal. and Strat.* **20**, 225–262 (2005).
9. Bond, D.P.G. & Wignall, P.B. Pyrite framboid study of marine Permian–Triassic boundary sections: A complex anoxic event and its relationship to contemporaneous mass extinction. *Geol. Soc. of Am. Bull.* **122**, 1265–1279; https://doi.org/10.1130/B30042.1 (2010).
10. Bond, D.P.G., Wignall, P.B. & Racki, G. Extent and duration of marine anoxia during the Frasnian–Famennian (Late Devonian) mass extinction in Poland, Germany, Austria and France. *Geol. Magazine* **141**, p. 173–193; https://doi.org/10.1017/S0016756804008866 (2004).
11. Boussaha, M., Thibault, N., and Stemmerik, L. Integrated stratigraphy of the late Campanian–Maastrichtian in the Danish Basin: revision of the Boreal calcareous nannofossil zonation. *Newslet. on Strat.* **49**, 337–360 (2012).
12. Bromley, R.G. Enhancement of visibility of structures in marly chalk: Modification of the Bushinsky oil technique. *Bull. of the Geol. Soc. of Denmark* **29**, 111–118 (1981).
13. Byrne, R.H., and Sholkovitz, E.R. Chapter 158 Marine chemistry and geochemistry of the lanthanides. In *Handbook on the Physics and Chemistry of Rare Earths* (eds Bünzli, J.-C.G. & Pecharsky, V.K.) **58**, 497–593 (1996).
14. Caldwell, R.L. & Dingle, H. Stomatopods. *Scientific American* **234**, 80–89 (1976).

15. Christensen, W.K. Palaeobiogeography of Late Cretaceous belemnites of Europe. *Paläontol. Zeitschrift* **50**, 113–129 (1976).

16. d'Orbigny, A. D. Mémoire sur les foraminifères de la Craie blanche du Bassin de Paris. *Mémoires de la Soc. Géol. de France* **4**, 1–51 (1840).

17. de Baar, H.J.W., Schijf, J. & Byrne, R.H. Solution chemistry of the rare earth elements in seawater. *European J.l of Solid State and Inorganic Chem.* **28**, 357–373 (1991).

18. de Vries, M.S. The role of feeding morphology and competition in governing the diet breadth of sympatric stomatopod crustaceans. *Biology letters* **13**; https://doi.org/10.1098/rsbl.2017.0055 (2017).

19. Dubicka, Z. & Peryt, D. Integrated biostratigraphy of Upper Maastrichtian chalk at Chełm (SE Poland). *Annal.Soc.s Geol. Pol.* **81**, 185–197 (2011).

20. Dubicka, Z. & Peryt, D. Latest Campanian and Maastrichtian paleoenvironmental changes: Implications from an epicontinental sea (SE Poland and western Ukraine). *Cret. Res.* **37**, 272–284 (2012).

21. Dubicka, Z., Wierzbowski, H., and Wierny, W. Oxygen and carbon isotope records of Upper Cretaceous foraminifera from Poland: vital and microhabitat effects. *Palaeogeography, Palaeoclimatology, Palaeoecology* **500**, 33–51: https://doi.org/10.1016/j.palaeo.2018.03.029 (2018).

22. Dzik, J. Origin of the Cephalopoda. *Acta Pal. Pol.* **26**, 161–191 (1981).

23. Ehrenberg, C. G. Eine weitere Erläuterung des Organismus mehrerer in Berlin lebend beobachteter Polythalamien der Nordsee. Bericht über die zu 65 Bekanntmachung geeigneten Verhandlugen der Köninglichen Preussischen Akademie der Wissenschaften zu Berlin 8–23 (1840).

24. Ekdale, A.A. & Bromley, R.G. Cretaceous chalk ichnofacies in northern Europe. *Geobios, Mémoire spécial* **8**, 201–204 (1984).

25. Ekdale, A.A. & Bromley, R.G. Analysis of composite ichnofabrics: An example in uppermost Cretaceous chalk of Denmark. *Palaios* **6**, 232–249 (1991).

26. Ellis, N.M. & Tobin, T.S. Evidence for seasonal variation in δ^{13}C and δ^{18}O profiles of *Baculites* and implications for growth rate. *Palaeontology* **62**, 583–598: https://doi.org/10.1111/pala.12416 (2019).

27. Fatheree, J. W., Harries, P. J. & Quinn, T. M. Oxygen and carbon isotopic "dissection" of *Baculites compressus* (Mollusca: Cephalopoda) from the Pierre Shale (upper Campanian) of South Dakota; implications for paleoenvironmental reconstructions. *Palaios* **13**, 376–385 (1998).

28. Ferguson, K., Macleod, K.G., Landman, N.H., and Sessa, J.A., 2019, Evaluating growth and ecology in baculitid and scaphitid ammonites using stable isotope sclerochronology. *Palaios* **34**, 317–329; https://doi.org/10.2110/palo.2019.005.

29. Fischer, A.G., and Fay, R.O. A spiny aptychus from the Cretaceous of Kansas. *Bull. of the State Geol. Survey of Kansas* **102**, 77–92 (1953).

30. Fraaije, R.H.B. The oldest *in situ* hermit crab from the Lower Cretaceous of Speeton, UK. *Palaeontology* **46**, 53–57 (2003).

31. Fraaije, R.H.B., Jagt, J.W.M., van Bakel, B.W.M., and Tshudy, D.M., 2020, A new early Late Cretaceous nephropid lobster (Crustacea, Decapoda) from Kazakhstan, entombed within an

ammonite body chamber. *Cret. Res.* **115**, 104552; https://doi.org/10.1016/j.cretres.2020.104552 (2020).

32. Fraaye, R.H.B. Late Cretaceous swimming crabs: radiation, migration, competition, and extinction. *Acta Geol. Pol.* **46**,269–278 (1996).
33. Fraaye, R.H.B., and Jäger, M. Decapods in ammonite shells: examples of inquilinism from the Jurassic of England and Germany. *Palaeontology* **38**, 63–75 (1995).
34. Geary, D.H., Allmon, W.D., and Reaka-Kudla, M.L., 1991, Stomatopod predation on fossil gastropods from the Plio-Pleistocene of Florida: *J. of Pal.* **65**, 355–360; https://doi.org/10.1017/S0022336000030341 (1991).
35. Guerra, Á., Rodríguez-Navarro, A.B., González, Á.F., Romanek, C.S., Álvarez-Lloret, P. & Pierce, G.J. Life-history traits of the giant squid *Architeuthis dux* revealed from stable isotope signatures recorded in beaks. *ICES J. of Marine Science* **67**,1425–1431; https://doi.org/10.1093/icesjms/fsq091 (2010).
36. Hagenow, F. von. Monographie der Rügen'schen KreideVersteinerungen. Abt. III - Mollusken, Neus Jahrbuch für Mineralogie. *Geognosie, Geologie und Petrefakten-Kunde* **1842**, 528–575 (1842).
37. Håkansson, E., Bromley, R. & Perch-Nielsen, K. Maastrichtian chalk of northwest Europe – a pelagic shelf sediment. In *Pelagic Sediments: on land and under the sea: Special Publications of the International Association of Sedimentologists* (eds Hsu, K.J. *et al.*) **1**, 211–233 (1974).
38. Hancock, J.M. Transatlantic correlations in the Campanian-Maastrichtian stages by eustatic changes of sea-level. *Geol. Soc.*, *Spec. Publ.*, **70**, 241–256 (1993).
39. Hansen, T. & Surlyk, F. Marine macrofossil communities in the uppermost Maastrichtian chalk of Stevns Klint, Denmark. *Palaeogeography*, *Palaeoclimatology*, *Palaeoecology* **399**, 323–344 (2014).
40. Hoffmann, R., Riechelmann, S., Ritterbush, K.A., Koelen, J., Lübke, N., Joachimski, M.M., Lehmann, J. & Immenhauser, A. A novel multiproxy approach to reconstruct the paleoecology of extinct cephalopods. *Gondwana Research* **67**, 64–81; https://doi.org/10.1016/j.gr.2018.10.011 (2019).
41. Janiszewska, K., Mazur, M., Machalski, M. & Stolarski, J. From pristine aragonite to blocky calcite: Exceptional preservation and diagenesis of cephalopod nacre in porous Cretaceous limestones. *PloS One* **13**, e0208598 (2018).
42. Jones, B. & Manning, D.A.C. Comparison of geochemical indices used for the interpretation of palaeoredox conditions in ancient mudstones. *Chem. Geol.* **111**, 111–129; https://doi.org/10.1016/0009-2541(94)90085-X (1994).
43. Kamber, B.S., Webb, G.E. & Gallagher, M. The rare earth element signal in Archaean microbial carbonate: information on ocean redox and biogenicity. *J. of the Geol. Soc.* **171**, 745–763; https://doi.org/10.1144/jgs2013-110 (2014).
44. Kauffman, E.G. Ecological reappraisal of the German Posidonienschiefer (Toarcian) and the staangant basin model. In Communities of the past. Hutchinson Ross Stroudsberg Pennsylvania (eds Gray, J. *et al.*) 311–381 (1981).
45. Keupp, H. Sublethal punctures in body chambers of Mesozoic ammonoids (forma *aegra fenestra* n. f.), a tool to interpret synecological relationships, particularly predator-prey interactions. *Paläont. Zeitschrift* **80**, 112–123; https://doi.org/10.1007/BF02988971 (2006).

46. Keutgen, N. A bioclast-based astronomical timescale for the Maastrichtian in the type area (southeast Netherlands, northeast Belgium) and stratigraphic implications: the legacy of P.J. Felder. *Netherlands J. of Geosc./Geol. en Mijnb.* **97**, 229–260; https://doi.org/10.1017/njg.2018.15 (2018).

47. Klompmaker, A.A., Waljaard, N.A. & Fraaije, R.H.B. Ventral bite marks in Mesozoic ammonoids. *Palaeogeography, Palaeoclimatology, Palaeoecology* **280**, https://doi.org/10.1016/j.palaeo.2009.06.013 (2009).

48. Kröger, B. Schalenverletzungen an jurassischen Ammoniten – ihre paläobiologische und paläoökologische Aussagefähigkeit. *Berliner Geowissenschaftliche Abhandlungen, Reihe E* **33**, 1–97 (2000).

49. Kruta, I. & Landman, N.H. Injuries on *Nautilus* jaws: implications for the function of ammonite aptychi. *The Veliger* **50,** 241–247 (2008).

50. Kruta, I., Landman, N.H. & Cochran, J.K. A new approach for the determination of ammonite and nautilid habitats. *PloS ONE* **9**, e87479; https://doi.org/10.1371/journal.pone.0087479 (2014).

51. Landman, N.H., Cobban, W.A. & Larson, N.L. Mode of life and habitat of scaphitid ammonoids. *Geobios* **45**, 87–98; https://doi.org/10.1016/j.geobios.2011.11.006 (2012).

52. Landman, N.H., Goolaerts, S., Jagt, J.W.M., Jagt-Yazykova, E.A., Machalski, M. & Yacobucci, M.M. Ammonite extinction and nautilid survival at the end of the Cretaceous. *Geology* **42**, 707–710 (2014).

53. Landman, N.H., Johnson, R.O., Garb, M.P., Edwards, L.E. & Kyte, F.T. Cephalopods from the Cretaceous/Tertiary boundary interval on the Atlantic Coastal Plain, with a description of the highest ammonite zones in North America. Part 3, Manasquan River Basin, Monmouth County, New Jersey. *Bull. of the Am. Mus. of Nat. Hist.* **303**, 122 (2007).

54. Larson, N.L. Predation and pathologies in the Late Cretaceous ammonite family Scaphitidae. *MAPS Digest Mid-Am. Pal. Soc.*, Macomb IL **26**, 1–23 (2002).

55. Larson, N.L. & Landman, N.H. Description of the lower jaws of *Baculites* from the Upper Cretaceous U.S. Western Interior. *Acta Geol. Pol.* **67**, 109–120 (2017).

56. Lauridsen, B.W., and Surlyk, F. Benthic faunal response to late Maastrichtian chalk-marl cyclicity at Rørdal, Denmark. *Palaeogeography*, *Palaeoclimatology*, *Palaeoecology* **269**, 38–53, https://doi.org/10.1016/j.palaeo.2008.07.001 (2008).

57. Lauridsen, B.W., Surlyk, F. & Bromley, R.G. Trace fossils of a cyclic chalk-marl succession; the upper Maastrichtian Rørdal Member, Denmark. *Cret. Res.* **32**, 194–202; https://doi.org/10.1016/j.cretres.2010.12.002 (2011).

58. Leckie, R.M., 1987, Paleoecology of mid-cretaceous planktonic foraminifera: a comparison of open ocean and epicontinental sea assemblages. *Micropaleontology* **33**, 164–176; https://doi.org/10.2307/1485491 (1987).

59. Łopuski, C., 1911, Przyczynki do znajomości fauny kredowej gub. Lubelskiej. *Sprawozd. z pos. Towarz. Nauk. Warszaw.* **4**, 104–110 (1911).

60. Lukeneder, A., Harzhauser, M., Müllegger, S., and Piller, W.E., 2010, Ontogeny and habitat change in Mesozoic cephalopods revealed by stable isotopes ($\delta^{18}O$, $\delta^{13}C$). *Earth and Planetary Sci. Letters* **296**, 103–114; https://doi.org/10.1016/j.epsl.2010.04.053 (2010).

61. Machalski, M. Late Maastrichtian and earliest Danian scaphitid ammonites in central Europe: taxonomy, evolution, and extinction. *Acta Pal. Pol.* **50**, 653–696 (2005).

62. Machalski, M. A new ammonite zonation for the Maastrichtian Stage in Poland. In *The Maastrichtian stage; the current concept* (eds Jagt, J.W.M. *et al.*). Workshop programme, abstracts and field guide, 40–44 (2012).

63. Machalski, M. Correlation of shell and aptychus growth provides insights into the palaeobiology of a scaphitid ammonite. *Palaeontology* **64**, 225–247; https://doi.org/10.1111/pala.12519 (2021).

64. Machalski, M., and Heinberg, C., 2005, Evidence for ammonite survival into the Danian (Paleogene) from the Cerithium Limestone at Stevns Klint, Denmark. *Bulletin of the Geological Society of Denmark* **52**, 97–111, https://doi.org/10.37570/bgsd-2005-52-08.

65. Machalski, M. & Malchyk, O. Durophagous predation on late Maastrichtian (Cretaceous) scaphitid ammonoids from Poland. In 10th International Symposium 'Cephalopods – Present and Past', Program and Abstracts. Münstersche Forschungen zur Geologie und Paläontologie 110, 77–78 (2018).

66. Machalski, M. & Malchyk, O. Relative bathymetric position of opoka and chalk in the Late Cretaceous European Basin. *Cret. Res.* **102**, 30–36, https://doi.org/10.1016/j.cretres.2019.05.007 (2019).

67. Machalski, M., Jagt, J. & Dubicka, Z. Additional records of scaphitid ammonites from the basal upper Maastrichtian (Upper Cretaceous) of eastern Poland. *Bull. de l'Institut Royal des Scien. Naturelles de Belgique* **78**, 261–269 (2008).

68. Machel, H. G., Mason, R.A., Mariano A.N. & Mucci A. Causes and emission of luminescence in calcite and dolomite. In *Luminescence Microscopy and Spectroscopy – Qualitative and quantitative applications* (eds Barker, C.E. & Kopp, O.C.) 9–25 (1991).

69. Machel, H.G. Application of cathodoluminescence to carbonate diagenesis. In *Cathodoluminescence in Geosciences* 271–301; https://doi.org/10.1007/978-3-662-04086-7_11 (2000).

70. Makowski, H. Problem of sexual dimorphism in ammonites. *Pal. Pol.* **12**, 1–92 (1962).

71. Mironenko, A. Sublethal injuries on the shells of Jurassic ammonoids from Central Russia. In Jurassic deposits of the southern part of the Moscow syneclise and their fauna (eds Rogov, M.A. & Zakharov, V.A). Transactions of the Geological Institute, GEOS, 183–208 (2017) (in Russian).

72. Mironenko, A., 2020, A hermit crab preserved inside an ammonite shell from the Upper Jurassic of central Russia: Implications to ammonoid palaeoecology. *Palaeogeography, Palaeoclimatology, Palaeoecology* **537**, 109397; https://doi.org/10.1016/j.palaeo.2019.109397 (2020).

73. Nederbragt, A.J., Erlich, R.N., Fouke, B.W. & Ganssen, G.M. Palaeoecology of the biserial planktonic foraminifer *Heterohelix moremani* (Cushman) in the late Albian to the middle Turonian Circum-North Atlantic. *Palaeogeography, Palaeoclimatology, Palaeoecology* **14**, 115–133, https://doi.org/10.1016/S0031-0182(98)00089-3 (1998).

74. Nestler, H. Die Rekonstruktion des Lebensraumes der Rügener Schreibkreide-Fauna (Unter-Maastricht) mit Hilfe der Paläoökologie und Paläobiologie. *Beiheft zur Zeitschrift Geol.* **49**, 1–147 (1965).

75. Neuser, R.D., Bruhn, F., Gotze, J., Habermann, D. & Richter, D.K. Kathodolumineszenz: Methodik und Anwendung. *Zentralblatt für Geol. und Pal.* **I**, 287–306 (1996).

76. Nozaki, Y. Rare earth elements and their isotopes in the ocean. In *Encyclopedia of Ocean Sciences* (eds Steele, J.H., *et al.*) 2354–2366 (Academic Press, 2001).

77. Parent, H., Westermann, G.E.G. & Chamberlain, J.A., Jr. Ammonite aptychi: Functions and role in propulsion. *Geobios* **47**, 45–55 (2014).

78. Peryt, D., 1980, Planktic foraminifera zonation of the Upper Cretaceous in the middle Vistula River valley, Poland. *Pal. Pol.* **41**, 1–123 (1980).

79. Pessagno, E.A. Upper Cretaceous planktonic foraminifera from the West Coastal Plain. *Palaeontogr. Amer.* **5**, 259–441 (1967).

80. Pether, J., 1995, *Belichnus* new ichnogenus, a ballistic trace on mollusc shells from the Holocene of the Benguela region, South Africa. *J. of Pal.* **69**, 171–181 (1995).

81. Radwański, A. The predation upon, and the extinction of, the latest Maastrichtian populations of the ammonite species *Hoploscaphites constrictus* (J. Sowerby, 1817) from the Middle Vistula Valley, Central Poland. *Acta Geol. Pol.* **46**, 117–136 (1996).

82. Rakociński, M., Pisarzowska, A., Janiszewska, K. & Szrek, P. Depositional conditions during the Lower Kellwasser Event (Late Frasnian) in deep-shelf Łysogóry basin of the Holy Cross Mountains (Poland). *Lethaia* **49**, 571–590; https://doi.org/10.1111/let.12167 (2016).

83. Reich, M. & Frenzel, P. Die Fauna und Flora der Rügener Schreibkreide (Maastrichtium, Ostsee). *Archiv für Geschiebekunde* **3**,73–284 (2002).

84. Rexfort, A. & Mutterlose, J. The role of biogeography and ecology on the isotope signature of cuttlefishes (Cephalopoda, Sepiidae) and the impact on belemnite studies: *Palaeogeography*, *Palaeoclimatology*, *Palaeoecology* **284**, 153–163; https://doi.org/10.1016/j.palaeo.2009.09.021 (2009).

85. Schneibnerovà, V. Implications of deep sea drilling in the Atlantic for studies in Australia and New Zealand. Some new views on Cretaceous and Cainozoic palaeogeography and biostratigraphy. *Search* **2**, 251–254 (1971).

86. Schulz, M.G. & Schmid, F. Das Ober-Maastricht von Hemmoor (N-Deutschland): Faunenzonen-Gliederung und Korrelation mit dem Ober–Maastricht von Dänemark und Limburg. *Newsletters on Strat.* **13**, 203–215 (1983).

87. Seilacher, A., 1993, Ammonite aptychi: how to transform a jaw into an operculum? *Am. J. of Sci.* **293**, 20–32 (1993).

88. Sessa, J.A., Larina, E., Knoll, K., Garb, M., Cochran, J.K., Huber, B.T., MacLeod, K.G. & Landman, N.H. Ammonoid habitat revealed via isotopic composition and comparisons with co-occurring benthic and planktonic organisms. *PNAS* **112**, 15562–15567: https://doi.org/10.1073/pnas.1507554112 (2015

89. Sowerby, J. The mineral conchology of Great Britain. Vol. 1, pls 1–9 (1812), pls 10–44 (1813), pls 45–78 (1814), pls 79–102 (1815); Vol. 2, pls 103–14 (1815), pls 115– 50 (1816), pls 151–86 (1817), pls 187–203 (1818); Vol. 3, pls 204–21 (1818), pls 222–53 (1819), pls 254–71 (1820), pls 272–306 (1821); Vol. 4, pls 307–18 (1821), pls 319–83 (1822) (London, 1812–1822).

90. Stevens, K., Mutterlose, J. &Wiedenroth, K. Stable isotope data (δ^{18}O, δ^{13}C) of the ammonite genus *Simbirskites*—implications for habitat reconstructions of extinct cephalopods. *Palaeogeography*, *Palaeoclimatology*, *Palaeoecology* **417**, 164–175; https://doi.org/10.1016/j.palaeo.2014.10.031 (2015).

91. Surlyk, F. Morphological adaptations and populationstructures of the Danish chalk brachiopods. Det Kongelige Danske Videnskabernes Selskab, *Biologiske Skrifter* **19**, 1–57 (1972).

100. Surlyk, F. & Birkelund, T. An integrated stratigraphical study of fossil assemblages from the Maastrichtian white chalk of northwestern Europe. In Concepts and methods of

biostratigraphy (eds Kauffman, E.G. & Hazel, J.E.) 257–281 (Dowden Hutchinson & Ross,1977).

92. Surlyk, F., Dons, T., Clausen, C.K. & Higham, J. Upper Cretaceous. In *The Millennium Atlas: petroleum geology of the central and northern North Sea* 213–233 (Geological Society of London, 2003).

93. Surlyk, F., Rasmussen, S.L., Boussaha, M., Schiøler, P., Schovsbo, N.H., Sheldon, E., Stemmerik, L. & Thibault, N.R. Upper Campanian-Maastrichtian holostratigraphy of the eastern Danish Basin. *Cret. Res*. 46, 232–256; https://doi.org/10.1016/j.cretres.2013.08.006 (2013).

94. Sweere, T., van den Boorn, S., Dickson, A.J. & Reichart, G.-J. Definition of new trace-metal proxies for the controls on organic matter enrichment in marine sediments based on Mn, Co, Mo and Cd concentrations. *Chem. Geol.* **441**, 235–245; https://doi.org/10.1016/j.chemgeo.2016.08.028 (2016).

95. Tagliavento, M., Lauridsen, B.W. & Stemmerik, L. Episodic dysoxia during Late Cretaceous cyclic chalk-marl deposition – evidence from framboidal pyrite distribution in the upper Maastrichtian Rørdal Mb., Danish Basin. *Cret. Res.* **106**, 104223; https://doi.org/10.1016/j.cretres.2019.104223 (2020).

96. Takeda, Y., Tanabe, K., Sasaki, T. & Landman, N.H. Durophagous predation on scaphitid ammonoids in the Late Cretaceous Western Interior Seaway of North America. *Lethaia* **49**, 28–42 (2016).

97. Tanabe, K., Kruta, I. & Landman, N.H. Ammonoid buccal mass and jaw apparatus. In *Ammonoid Paleobiology: From macroevolution to paleogeography* (eds Klug. C. *et al.*) 439–494 (Springer, 2015).

98. Thibault, N., Harlou, R., Schovsbo, N., Schiøler, P., Minoletti, F., Galburn, B., Lauridsen, B.W., Sheldon, E., Stemmerik, L. & Surlyk, F. Upper Campanian-Maastrichtian nannofossil biostratigraphy and high-resolution carbon-isotope stratigraphy of the Danish Basin: Towards a standard $\delta^{13}C$ curve for the Boreal Realm. *Cret. Res.* **33**, 72–90; https://doi.org/10.1016/j.cretres.2011.09.001 (2012).

99. Trauth, F. Aptychenstudien I-VIII. *Annalen des Naturhistorischen Museums in Wien* **41**, 171–259 (1927); **42**, 121–193 (1928); **44**, 329–411 (1930); **45**, 17–136 (1931); **47**, 127–145 (1936).

100. Tribovillard, N., Algeo, T.J., Lyons, T. & Riboulleau, A. Trace metals as paleoredox and paleoproductivity proxies: An update. *Chem. Geol.* **232**, 12–32, https://doi.org/10.1016/j.chemgeo.2006.02.012 (2006).

101. Walaszczyk, I., Dubicka, Z., Olszewska-Nejbert, D. & Remin, Z. Integrated biostratigraphy of the Santonian through Maastrichtian (Upper Cretaceous) of extra-Carpathian Poland. *Acta Geol. Pol*. **66**, 321–358; https://doi.org/10.1515/agp-2016-0016 (2016).

102. Wignall, P.B. & Newton, R. Pyrite framboid diameter as a measure of oxygen deficiency in ancient mudrocks. *Am. J. of Sci.* **298**, 537–552; https://doi.org/10.2475/ajs.298.7.537 (1998).

103. Wignall, P.B. & Simms, M.J. Pseudoplankton. *Palaeontology* **33**, 359–378 (1990).

104. Wilkin, R.T., Arthur, M., and Dean, W. History of water-column anoxia in the Black Sea indicated by pyrite framboid size distributions. *Earth and Planetary Science Letters* **148**, 517–525, https://doi.org/10.1016/S0012-821X(97)00053-8 (1997).

105. Wilkin, R.T., Barnes, H.L. & Brantley, S.L. The size distribution of framboidal pyrite in modern sediments: An indicator of redox conditions. *Geochimica et Cosmochimica Acta* **60**, p. 3897–3912; https://doi.org/10.1016/0016-7037(96)00209-8 (1996).

106. Wilmsen, M. & Niebuhr, B. High-resolution Campanian-Maastrichtian carbon and oxygen stable isotopes of bulk-rock and skeletal component: Palaeoceanographic and palaeoenvironmental implications for the Boreal shelf sea. *Acta Geol. Pol.* **67**, 47–74; https://doi.org/10.1515/agp-2017-0004 (2017).

107. Witwicka, E. Stratygrafia mikropaleontologiczna kredy górnej wiercenia w Chełmie. *Biul. Inst. Geol.* **121**, 177–267 (1958).

108. Zhang, K.-J., Li, Q.-H., Yan, L.-L., Zeng, L., Lu, L., Zhang, Y.-X., Hui, J., Jin, X. & Tang, X.-C., 2017, Geochemistry of limestones deposited in various plate tectonic settings. *Earth-Science Reviews* **167**, 27–46, https://doi.org/10.1016/j.earscirev.2017.02.003 (2017).
